# Intracoronary Vasoactive Therapy for No-Reflow During Primary PCI: A Network Meta-Analysis of Randomized Trials

**DOI:** 10.1016/j.jacadv.2026.102599

**Published:** 2026-02-25

**Authors:** Federico Oliveri, Lorenzo Tua, Luca Raone, Francesco Maria Sparasci, Marco Ferlini, Alessandro Mandurino-Mirizzi, Leonardo De Luca, Fatih Arslan, Brian O. Bingen, Jose’ M. Montero-Cabezas

**Affiliations:** a Department of Cardiology, Leiden University Medical Center, Leiden, The Netherlands; b Cardiology Unit, Fondazione IRCCS Policlinico San Matteo, Pavia, Italy; c Division of Cardiology, “Vito Fazzi” Hospital, Lecce, Italy; d Department of Experimental Medicine (DiMeS), University of Salento, Lecce, Italy

**Keywords:** adenosine epinephrine, (adenosine OR epinephrine OR nitroprusside OR verapamil) AND no-reflow, nitroprusside, no-reflow, verapamil

## Abstract

**Background:**

The no-reflow phenomenon frequently complicates primary percutaneous coronary intervention and is associated with adverse outcomes. Evidence supporting a specific pharmacological treatment is limited.

**Objectives:**

The aim of this study was to compare commonly used intracoronary medications for the treatment of the no-reflow phenomenon.

**Methods:**

We performed a systematic review and frequentist network meta-analysis of randomized controlled trials comparing intracoronary adenosine, epinephrine, nitroprusside, and verapamil administered during primary percutaneous coronary intervention for the treatment of the no-reflow phenomenon (PROSPERO CRD420251102877). The primary efficacy endpoint was restoration of TIMI flow grade 3 flow at the end of the procedure. Secondary endpoints included ST-segment resolution, major adverse cardiovascular events, and all-cause mortality.

**Results:**

Thirteen studies, for a total of 1,674 patients, were included in the analysis. Both epinephrine (OR: 2.81; 95% CI: 1.72-4.58) and verapamil (OR: 2.84; 95% CI: 1.63-4.95) were associated with significantly higher odds of achieving final TIMI flow grade 3 compared with control (τ^2^ = 0; I^2^ = 0%). Meta-regression found no confounding effect from intraprocedural glycoprotein IIb/IIIa inhibitors or mechanical thrombectomy. For ST-segment resolution, significant benefits were observed with epinephrine (OR: 4.30; 95% CI: 2.19-8.45), verapamil (OR: 2.85; 95% CI: 1.64-4.96), and adenosine (OR: 1.38; 95% CI: 1.04-1.84), compared with control (τ^2^ = 0; I^2^ = 0%). None of the therapies reduced major adverse cardiovascular events or mortality, although the analysis was underpowered.

**Conclusions:**

In this network meta-analysis, intracoronary verapamil and epinephrine were associated with improvements in final TIMI flow grade 3 compared with control. Further studies are needed to clarify optimal agent selection.

## Central Illustration

**Figure:**
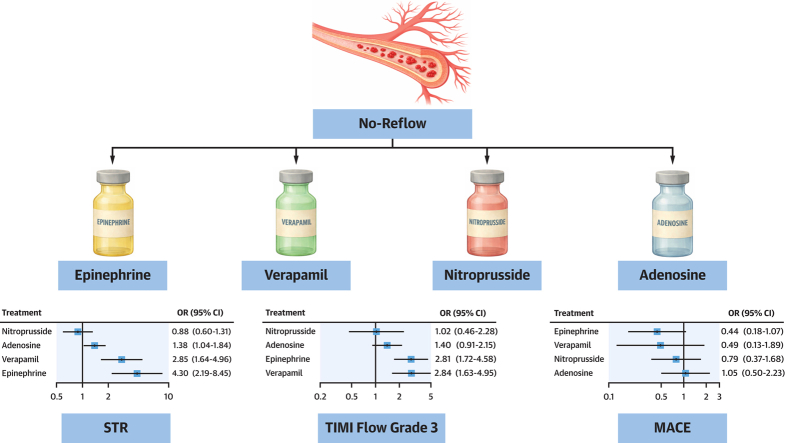

Although primary percutaneous coronary intervention (PCI) represents the cornerstone of reperfusion therapy in ST-segment elevation myocardial infarction, successful restoration of epicardial coronary patency does not invariably translate into effective tissue-level perfusion.^1^ The no-reflow phenomenon, defined as persistent myocardial hypoperfusion in the absence of residual epicardial mechanical obstruction, occurs in a substantial proportion of patients and is associated with larger infarct size, worse left ventricular remodeling and increased mortality.^2^, ^3^, ^4^, ^5^, ^6^ Its incidence varies widely depending on the diagnostic modality employed, ranging from 5 to 25%.^7^, ^8^, ^9^ The underlying mechanisms of coronary no-reflow are multifactorial and involve a dynamic interplay between mechanical obstruction by microthrombi or plaque debris, endothelial dysfunction, ischemia-reperfusion injury, vasoconstriction, and inflammatory microvascular damage.^2^^,^^3^ Recognition of its prognostic relevance has led to the development of multiple pharmacologic strategies aimed at promoting microvascular reperfusion in the acute setting.^10^ Although several procedural (eg, direct stenting, thrombectomy, or distal embolic protection for graft) and pharmacologic strategies (eg intracoronary vasodilators, glycoprotein (GP) IIb/IIIa inhibitors, and intracoronary thrombolytics) have been proposed to manage the no-reflow phenomenon, none has consistently demonstrated superiority over the others.^11^^,^^12^ Recent evidence has shown that intracoronary administration of agents such as adenosine, verapamil, epinephrine, and nitroprusside may play a significant role in restoring coronary flow. However, the lack of standardized dosing protocols and the frequent use of drug combinations, even among different operators within the same center, underscore the variability in current practice and the need for more robust evidence. To address this gap, we conducted a network meta-analysis (NMA) of randomized controlled trials (RCTs) evaluating the efficacy of adenosine, verapamil, epinephrine, or nitroprusside used in the treatment of the no-reflow phenomenon during primary PCI.

## Methods

This systematic review and NMA was conducted in accordance with the Preferred Reporting Items for Systematic Reviews and Meta-Analyses guidelines.^13^ The full study protocol was registered in the PROSPERO (CRD420251102877).

### Search strategy and data extractions

Four independent reviewers (F.O., L.T., L.R., and F.S.) performed the initial screening of titles and abstracts to identify potentially eligible studies, each one for 1 of the drugs included in our paper (adenosine, epinephrine, verapamil, and nitroprusside). Primary keywords were “adenosine” "epinephrine", “nitroprusside”, “verapamil”,” no-reflow”, “(adenosine OR epinephrine OR nitroprusside OR verapamil) AND no-reflow”. They systematically searched the Pubmed, Scopus, Cochrane, and EMBASE databases starting on February 21, 2025, and finishing on May 30, 2025. In case of disagreement, shared decisions were with other coauthors (M.F. and A.M.M.). Data were therefore extracted independently using a standardized recording tool to document the study setting and design, year of publication, number of study participants, country of origin, participant clinical characteristics, and study outcomes. The Kappa statistic was used to assess the inter-rater reliability of the reviewers.^14^

### Inclusion criteria

The screening focused on studies enrolling patients with acute coronary syndromes undergoing primary PCI. Eligible interventions included intraprocedural intracoronary administration of adenosine, epinephrine, nitroprusside, verapamil, or placebo. Only RCTs were considered for inclusion. Studies were excluded if they involved combination therapies using 2 or more of the aforementioned agents, included patients with stable coronary artery disease, or used intravenous administration of the study drug. Intracoronary medications could be administered at different procedural stages, either before or after balloon inflation or stenting, but never as a preventive strategy before guidewire advancement. We adopted an inclusive definition of “no-reflow,” encompassing both the classical form and persistent no-flow (TIMI flow grade 0) observed after guidewire passage but before balloon inflation or stenting.^15^ This broader definition was selected to reflect the clinical continuum of impaired myocardial perfusion during PCI, as both phenomena are associated with adverse outcomes and are frequently reported under the umbrella term “no-reflow” in the literature. The use of thrombectomy or GP IIb/IIIa inhibitors was recorded for each study as potential confounders.

### Endpoint

The primary efficacy endpoint was the restoration of TIMI flow grade 3 at the end of the procedure. Secondary efficacy endpoints included the achievement of TIMI flow grade 2 to 3 and ST-segment resolution (STR) on electrocardiogram (>70% reduction from baseline). Exploratory clinical outcomes were major adverse cardiovascular events (MACE) and all-cause mortality.

### Quality assessment

Two authors (F.O. and L.T.) assessed the risk of bias of all considered RCTs according to the Cochrane Collaboration's tool and Cochrane RoB-2.^16^ In case of discrepancy, a consensus with a third author (A.M.M.) was made the final evaluation. We explored the potential for publication bias by visual inspection of funnel plots.

### Data analysis and synthesis

We conducted a NMA using both frequentist and Bayesian frameworks to evaluate treatments for no-flow in primary PCI. A random-effects model was applied to account for between-study variability. Treatment effects were expressed as ORs for binary outcomes along with their corresponding 95% CIs (frequentist) or 95% credible intervals (CrIs) (Bayesian). Network plots were generated for each outcome to illustrate the geometry of the evidence and the connectivity between interventions. Pooled-effect estimates were summarized using forest plots, whereas treatment rankings were presented using surface under the cumulative ranking curve values and ranking probability plots. Between-study heterogeneity was quantified using the I^2^ statistic and between-study variance (τ^2^). Small study effects and potential publication bias were explored using funnel plots. Bayesian network meta-analyses were conducted using random-effects models. Weakly informative priors were used to minimize prior influence on treatment effect estimates. Treatment effects were assigned normal priors centered at zero with large variance, reflecting no assumed effect a priori, whereas between-study heterogeneity was modeled using a half-normal prior. Posterior distributions were estimated using Markov chain Monte Carlo methods, and model convergence was assessed using the standard diagnostic criteria. Statistical incoherence was assessed globally using the design-by-treatment interaction model and locally through node-splitting analyses. Transitivity was assessed by evaluating the comparability of study populations, clinical context, outcome definitions, and control interventions across trials, as well as the distribution of key procedural characteristics. A contribution matrix was generated for the primary outcome to quantify the relative contribution of each direct comparison to the final network estimates. Prespecified sensitivity analyses were performed to evaluate the robustness of the findings, including leave-one-out analysis and the exclusion of studies at a higher risk of bias for our primary outcome. A meta-regression analysis was conducted to explore the influence of potential effect modifiers, including age, sex, GP IIb/IIIa inhibitor use, and mechanical thrombectomy on the final TIMI flow grade 3. Frequentist analyses were conducted using the netmeta, whereas Bayesian analyses and visualizations were performed using BUGSnet. Additional outputs, such as surface under the cumulative ranking curve rankings and network geometry plots, were also created using MetaInsight (version 6.0.1).

### Ethical considerations

Institutional review board or ethics committee approval was not required for this study because it was based exclusively on data from previously published RCTs and did not involve the collection or analysis of individual patient-level data.

## Results

**Figure 1:**
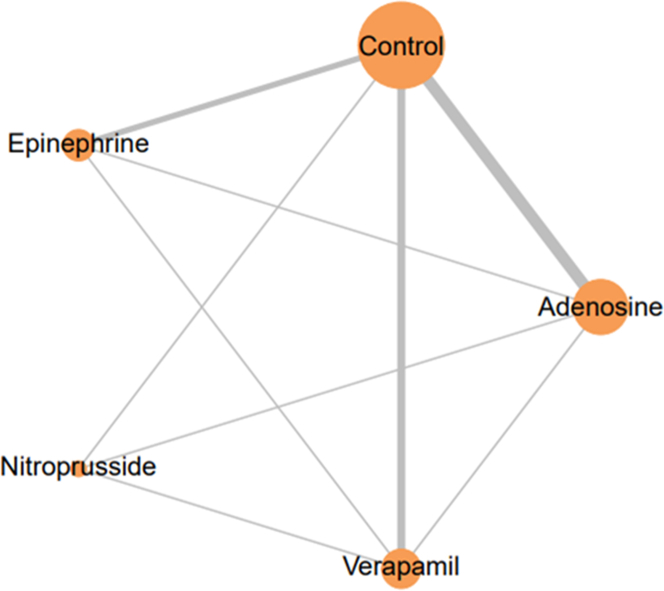
**Network Plots of Treatment Comparisons** Each node represents an individual treatment, and lines between nodes indicate direct comparisons available in the included studies. The size of the nodes and the thickness of the lines correspond to the number of studies and comparisons, respectively.

**Figure 2:**
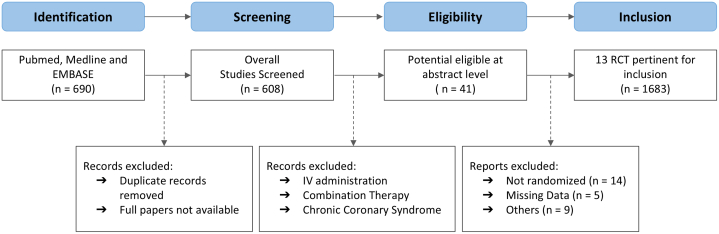
**Network Plots of Treatment Comparisons** IV = intravenous; RCT = randomized controlled trial.

A total of 680 records were identified in the preliminary search. A total of 13 studies were used for data extraction and synthesis for our NMA.^17^, ^18^, ^19^, ^20^, ^21^, ^22^, ^23^, ^24^, ^25^, ^26^, ^27^, ^28^, ^29^ Overall, 1,683 patients were included in our study. A total of 516 were treated with intracoronary adenosine, 196 with epinephrine, 189 with nitroprusside, 139 with verapamil, and 554 with control. Network Plot is shown in Figure 1, whereas the Preferred Reporting Items for Systematic Reviews and Meta-Analyses flow diagram in Figure 2. Studies and baseline characteristics are detailed in Table 1, Table 2, Table 3, respectively.

**Table 1:** Studies Characteristics

First Author	Journal	Country	Indication	Treatment	Control	Sample	Timing	Follow-up
Desmet et al^17^	European Heart Journal	Belgium	STEMI	Adenosine (4 mg)	0.9% NaCl	A: 56 C: 54	Before PCI	12 months
Garcia-Dorado et al^18^	International Journal of Cardiology	Spain	STEMI	Adenosine (4.5 mg)	0.9% NaCl	A: 100 C: 97	Before PCI	6 months
Naghshtabrizi et al^19^	Coronary Artery Disease	Iran	STEMI	Adenosine (40 ug + 40 ug)	0.9% NaCl	A 52 C: 52	First dose: after crossing Second dose: after stenting	30 days
Niccoli et al^2^^,^^3^^,^^21^^,^^30^	JACC Cardiovascular Intervention	Italy	STEMI	Adenosine (120 ug + 2 mg) Nitroprusside (60 ug + 100 ug)	0.9% NaCl	A: 80 N: 80 C: 80	Before PCI	30 days
Petronio et al^20^	American Heart Journal	Italy	pPCI	Adenosine	standard care^a^	A: 30 C: 30	Before PCI	6 months
Nazir et al^29^	European Heart Journal	United Kingdom	STEMI	Adenosine (1 mg + 1 or 2 mg) Nitroprusside: (250 ug + 250 ug)	standard care^b^	A: 82 N: 79 C: 86	First dose: before PCI Second dose: after stenting	6 months
Ryabov et al^22^	American Journal of Cardiology	Russia	STEMI	Epinephrine 100 mcg	standard care^c^	E: 45 C: 45	After failure of previous treatments	before hospital discharge
Abdelaziz et al^28^	Coronary Artery Disease	United Kingdom	pPCI	Nitroprusside (60 ug + 120 ug) Verapamil (250 ug + 500 ug)	Nothing	N: 30 V: 30	After lesion crossing	30 days
Faruk Akturk et al^26^	Minerva Cardioangiol.	Turkey	pPCI	Adenosine 240 ug Verapamil 1.5 mg	0.9% NaCl	A: 16 V: 15 C: 15	After PCI	6 months
Huang et al^27^	American Heart Journal	China	pPCI	Verapamil 200 ug bolus repeatable (max 2 gr)	NTG 200 ug bolus repeatable (max 1 gr)	V: 34 C: 34	After PCI	30 days
Taniyama et al^25^	JACC	Japan	pPCI	Verapamil 0.5 mg	Nothing	V: 20 C: 20	After PCI	24-25 days
Qiao et al^31^	Chinese Journal of Interventional Cardiology	China	pPCI	Verapamil 200ug	0.9% NaCl	V: 47 C: 44	After PCI	3 months
Yassin et al^23^	Cardiology & Vascular Research	Egypt	pPCI	Epinephrine 100 ug Verapamil 200 ug	Nothing	E: 40 V: 40 C: 40	After lesion crossing	30 days
Khan et al^24^	Circulation Cardiovasc Interv.	Pakistan	ACS	Adenosine 60- 1,000 μg Epinephrine 100-600 mcg	Nothing	A: 100 E: 101	After PCI	30 days

A = adenosine; ACS = acute coronary syndrome; C = control; E = epinephrine; N = nitroprusside; NTG = nitroglycerin; PCI = percutaneous coronary intervention; pPCI = primary percutaneous coronary intervention; STEMI = ST-elevation myocardial infarction; V = verapamil.

^a^Stenting only; ^b^Standard PPCI alone; ^c^At least 1 among nitroglycerin, adenosine, papaverine, glycoprotein IIB/IIIA inhibitors, and thrombectomy.

**Table 2:** Baseline Characteristics

First Author	Age (years)	Female (%)	Thrombectomy (%)	GP IIb-IIIa (Inhibitors)	TIMI 0-1 (Baseline)	MACE Definition
Desmet et al^17^	A: 61 ± 12 C: 61 ± 11	A: 21% C: 15%	A: NA C: NA	A: 91% C: 93%	A: 80% C: 63%	All-cause mortality and functional status (NYHA functional class)
Garcia-Dorado et al^18^	A: 60 ± 12 C: 59 ± 14	A: 15% C: 12%	A: 73% C: 77%	A: 63% C: 65%	A: 100% C: 100%	All-cause mortality
Naghshtabrizi et al^19^	A: NA C: NA	A: NA C: NA	A: NA C: NA	A: 0% C: 0%	A: 100% C: 100%	All-cause mortality
Niccoli et al^2^^,^^3^^,^^21^^,^^30^	A: 63 ± 11 N: 63 ± 10 C: 64 ± 13	A: 23% N: 26% C: 26%	A: 100% N: 100% C: 100%	A: 100% N: 100% C: 100%	A: 100% N: 100% C: 100%	Cardiac mortality, MI, HF, and TLR
Petronio et al^20^	A: 56 ± 14 C: 61 ± 13	A: 17% C: 13%	A: NA C: NA	A: NA C: NA	A: 100% C: 100%	All-cause mortality and HF
Nazir et al^29^	A: 58 ± 13 N: 61 ± 13 C: 60 ± 11	A: 21% N: 16% C: 14%	A: 99% N: 95% C: 93%	A: NA N: NA C: NA	A: 99% N: 91% C: 97%	All-cause mortality, MI, HF, and TLR
Ryabov et al^22^	E: 65 ± 10 C: 63 ± 12	E: 33% C: 22%	E: 33% C: 51%	E: 33% C: 51%	E: 26% C: 16%	Cardiovascular mortality, MI, and HF
Abdelaziz et al^28^	N: 53 ± 9 V: 53 ± 8	N: 33% V: 33%	N: 0% V: 0%	N: 0% V: 0%	N: 100% C: 100%	Cardiovascular mortality, MI, HF, and TVR
Faruk Akturk et al^26^	A: 56 ± 10 V: 59 ± 14 C: 58 ± 13	A: 25% V: 13% C: 13%	A: 0% V: 0% C: 0%	A: 100% V: 100% C: 100%	A: 94% V: 93% C: 100%	None
Huang et al^27^	V: 65 ± 13 C: 66 ± 12	V: 24% C: 24%	V: 32% C: 38%	V: 88% C: 100%	V: 82% C: 88%	All-cause mortality, MI, and TVR
Taniyama et al^25^	V: 61 ± 11 C: 67 ± 14	V: 25% C: 20%	V: NA C: NA	V: NA C: NA	V: 100% C: 100%	None
Qiao et al^31^	V: 61 ± 12 C: 64 ± 11	V: NA C: NA	V: NA C: NA	V: NA C: NA	V: NA C: NA	Cardiac mortality, MI, HF, and recurrent angina
Yassin et al^23^	E: 53 ± 10 V: 56 ± 9 C: 56 ± 10	E: 20% V: 20% C: 23%	E: 83% V: 95% C: 80%	E: NA V: NA C: NA	E: 93% V: 95% C: 93%	All-cause mortality, MI, HF, TVR, and TLR
Khan et al^24^	A: 57 ± 11 E: 57 ± 12	A: 24% E: 31%	A: NA E: NA	A: 20% E: 24%	A: 63% E: 59%	MI, TVR, cerebrovascular accident, and HF

GP = glycoprotein; HF = heart failure; MACE = major adverse cardiovascular effect; MI = myocardial infarction; NA = not available; TLR = target lesion revascularization; TVR = target vessel revascularization; other abbreviations as in Table 1.

**Table 3:** Procedural and Clinical Outcomes

First Author	Final TIMI Flow Grade ≥II	Final TIMI Flow Grade III	Final cTFC (frames)	Final MBG 2-3	STR	MACE	Overall Mortality	Post-LV-EF (%)	VT/VF	3° AVB	Hypotension
Desmet et al^17^	A: 96% C: 96%	A: 80% C: 87%	A: 34.1 ± 25.2 C: 30.5 ± 19.6	A: 63% C: 59%	A: 45% C: 41%	A: 4% C: 4%	A: 4% C: 4%	A: 47.6 ± 9.5 C: 51.3 ± 8.7	A: 4% C: 6%	A: 18% C: 7%	A: NA C: NA
Garcia-Dorado et al^18^	A: 99% C: 99%	A: 94% C: 88%	A: NA C: NA	A: 82% C: 82%	A: 66% C: 57%	A: NA C: NA	A: 3% C: 1%	A: 49.5 ± 10.4 C: 49.4 ± 10.2	A: 2% C: 1%	A: 2% C: 0%	A: NA C: NA
Naghshtabrizi et al^19^	A: 96% C: 88%	A: 85% C: 63%	A: NA C: NA	A: NA C: NA	A: 17% C: 17%	A: NA C: NA	A: 4% C: 8%	A: 40.1 ± 11.9 C: 42.9 ± 7.7	A: 10% C: 21%	A: NA C: NA	A: NA C: NA
Niccoli et al^2^^,^^3^^,^^21^^,^^30^	A: 91% N: 90% C: 90%	A: 91% N: 90% C: 90%	A: NA N: NA C: NA	A: NA N: NA C: NA	A: 71% N: 54% C: 51%	A: 10% N: 7% C: 20%	A: 3% N: 3% C: 4%	A: NA N: NA C: NA	A: 3% N: 3% C: 3%	A: 13% N: 1% C: 3%	A: 6% N: 15% C: 9%
Petronio et al^20^	A: 93% C: 87%	A: 93% C: 87%	A: 16 ± 12 C: 23 ± 11	A: NA C: NA	A: 43% C: 53%	A: 7% C: 3%	A: 7% C: 3%	A: 47 ± 10 C: 48 ± 6	A:13% C: 13%	A: NA C: NA	A: NA C: NA
Nazir et al^29^	A:NA N: NA C: NA	A: NA N: NA C: NA	A: NA N: NA C: NA	A: NA N: NA C: NA	A: 68% N: 61% C: 65%	A: 13% N: 5% C: 2%	A: 1% N: 1% C: 0%	A: 43.2 + 7.9 N: 43.9 + 6.5 C: 45.7 + 8.0	A: 6% N: 4% C: 6%	A: 11% N: 4% C: 12%	A: 6% N: 4% C: 7%
Ryabov et al^22^	E: 56% C: 29%	E: 56% C: 29%	E: NA C: NA	E: NA C: NA	E: 78% C: 36%	E: 11% C: 22%	E: 7% C: 9%	E: 51.0 ± 8.6 C: 47.0 ± 8.8	E: 9% C: 7%	E: 0% C: 2%	E: NA C: NA
Abdelaziz et al^28^	N: 73% V: 90%	N: 73% V: 90%	N: 26.9 ± 4.5 V: 20.1 ± 3.6	N: 60% V: 87%	N: 17% V: 40%	N: 7% V: 3%	N: NA V: NA	N: 40.5 ± 4.7 V: 42.6 ± 4.9	N: NA V: NA	N: NA V: NA	N: 20% V: 3%
Faruk Akturk et al^26^	A: 69% V: 80% C: 40%	A: 6% V: 27% C: 0%	A: 71 ± 46 V: 52 ± 48 C: 71 ± 37	A: NA V: NA C: NA	A: 6% N: 7% C: 3%	A: NA N: NA C: NA	A: 3% N: 2% C: 2%	A: 41 ± 9.7 N: 42.1 ± 9.9 C: 39.9 ± 9.4	A: NA N: NA C: NA	A: NA N: NA C: NA	A: NA N: NA C: NA
Huang et al^27^	V: 85% C: 53%	V: 85% C: 53%	V: 28.4 ± 10.2 C: 42.4 ± 17.9	V: NA C: NA	V: 56% C: 29%	V: 9% C: 12%	V: 3% C: 9%	V: 57 ± 8.9 C: 58.1 ± 8.7	V: NA C: NA	V: 15% C: 0%	V: NA C: NA
Taniyama et al^25^	V: 85% C: 75%	V: 85% C: 75%	V: NA C: NA	V: NA C: NA	V: NA C: NA	V: NA C: NA	V: NA C: NA	V: 48 ± 11 C: 47 ± 9	V: NA C: NA	V: NA C: NA	V: NA C: NA
Qiao et al^31^	V: 100% C: 91%	V: NA C: NA	V: 27.1 ± 14.2 C: 39.0 ± 23.8	V: 91% C: 75%	V: NA C: NA	V: 15% C: 18%	V: NA C: NA	V: 63.4 ± 8.2 C: 63.5 ± 10.3	V: NA C: NA	V: NA C: NA	V: NA C: NA
Yassin et al^23^	E: 65% V: 63% C: 53%	E: 65% V: 63% C: 53%	E: NA V: NA C: NA	E: 85% V: 88% C: 33%	E: 83% V: 80% C: 65%	E: 1% V: 0% C: 0%	E: NA V: NA C: NA	E: 46.9 ± 9.3 V: 43.9 ± 7.1 C: 46.4 ± 7.8	E: 5% V: 0% C: 0%	E: NA V: NA C: NA	E: NA V: NA C: NA
Khan et al^24^	A: 78% E: 90%	A: 78% E: 90%	A: 24 ± 8.4 E: 26.6 ± 9.2	A: 56% E: 46%	A: NA E: NA	A: 6% E: 4%	A: 6% E: 4%	A: NA E: NA	A: 0% E: 3%	A: NA E: NA	A: NA E: NA

AVR = atrio-ventricular block; cTFC = Corrected Thrombolysis In Myocardial Infarction Frame Count; LV-EF = left ventricle ejection fraction; MBG = Myocardial blush grade; STR = ST-segment resolution; VF = ventricular fibrillation; VT = ventricular tachycardia; other abbreviations as in Tables 1 and 2.

### TIMI flow grade 3

**Figure 3:**
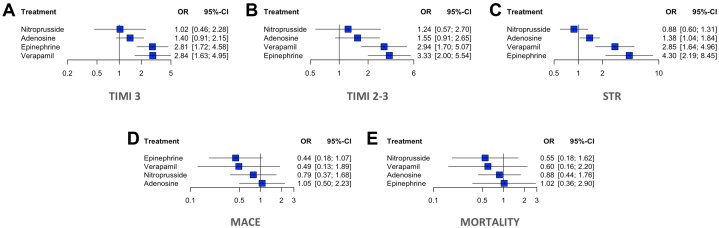
**Forest Plots of Primary and Secondary Outcomes Compared to Control** (A) TIMI flow grade 3; (B) TIMI flow grade 2 to 3; (C) ST-segment resolution (STR); (D) major adverse cardiovascular events (MACE); and (E) overall mortality. Each plot shows the effect estimates and 95% CIs for each treatment compared to control. Heterogeneity: TIMI flow grade 3 (τ^2^ = 0; I^2^ = 0%); TIMI flow grade 2 to 3 (τ^2^ = 0; I^2^ = 0%), STR (τ^2^ = 0; I^2^ = 0%), MACE (τ^2^ = 0; I^2^ = 0%), and mortality (τ^2^ = 0.15; I^2^ = 21.4%).

**Figure 4:**
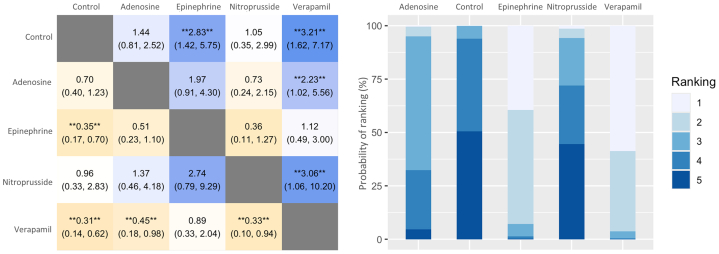
**League Plots and Ranking Plots for Each Outcome** The league plots (left) display head-to-head comparisons between treatments. The ranking plots (right) summarize the overall probability of each treatment being the most effective in achieving final TIMI flow grade 3.

Both epinephrine (OR: 2.81; 95% CI: 1.72-4.58) and verapamil (OR: 2.84; 95% CI: 1.63-4.95) were associated with significantly higher odds of achieving final TIMI flow grade 3 compared with control. In contrast, neither adenosine (OR: 1.40; 95% CI: 0.91-2.15) nor nitroprusside (OR: 1.02; 95% CI: 0.46-2.28) showed a statistically significant difference vs control (Figure 3). Estimated between-study heterogeneity was negligible (τ^2^ = 0; I^2^ = 0%). Findings were consistent in the Bayesian analysis (Supplemental Figure 1). League-plot and ranking-plot analyses indicated that verapamil had the highest probability of achieving final TIMI flow grade 3 (Figure 4).

### TIMI flow grade 2 to 3

In the frequentist analysis, all intracoronary agents were associated with numerically higher odds of achieving TIMI 2 to 3 flow restoration compared with control; however, only epinephrine (OR: 3.33; 95% CI: 2.00-5.54) and verapamil (OR: 2.94; 95% CI: 1.70-5.07) reached statistical significance (Figure 3). Estimated between-study heterogeneity was negligible (τ^2^ = 0; I^2^ = 0%). Findings were consistent in the Bayesian analysis (Supplemental Figure 2).

### ST-segment resolution

In the frequentist analysis, STR was significantly improved with epinephrine (OR: 4.30; 95% CI: 2.19-8.45), verapamil (OR: 2.85; 95% CI: 1.64-4.96), and adenosine (OR: 1.38; 95% CI: 1.04-1.84) compared with control. Estimated between-study heterogeneity was negligible (τ^2^ = 0; I^2^ = 0%) (Figure 3). However, in the Bayesian analysis, only epinephrine (OR: 4.43; 95% CrI: 2.14-9.78) and verapamil (OR 2.92; 95% CrI: 1.59-5.37) remained significantly associated with STR (Supplemental Figure 3).

### Adverse events

In the frequentist NMA evaluating the effect of intracoronary drugs on MACE, epinephrine was associated with the greatest numerical reduction in risk compared to control (OR: 0.43; 95% CI: 0.17-1.07), followed by verapamil (OR: 0.55; 95% CI: 0.14-2.14) and nitroprusside (OR: 0.80; 95% CI: 0.37-1.70), although none of these comparisons reached statistical significance (Figure 3). Between-study heterogeneity was low with τ^2^ = 0.15 and I^2^ = 21.4%. Results were consistent in the Bayesian analysis (Supplemental Figure 4).

### Mortality

None of the evaluated agents demonstrated a statistically significant reduction in mortality compared with control. In the frequentist model, there was a nonsignificant trend toward lower mortality with adenosine, nitroprusside (OR: 0.55; 95% CI: 0.18-1.62), and verapamil (OR: 0.60; 95% CI: 0.16-2.20). Estimated between-study heterogeneity was negligible (τ^2^ = 0; I^2^ = 0%) (Figure 3). The Bayesian model showed a similar neutral effect (Supplemental Figure 5).

### Quality assessment

Risk of publication bias and the quality assessment are presented in Supplemental Figures 12 and 13. Most trials were judged as having low risk or some concerns across key domains, with only a few studies presenting a high risk in specific areas such as missing outcome data or deviations from intended interventions (Supplemental Figure 13). Sensitivity analyses excluding higher-risk studies did not materially change the results for the primary endpoint, supporting the robustness of the main findings (Supplemental Figures 27 to 31).

### Sensitivity analysis

In the leave-one-out analysis, both epinephrine and verapamil remained associated with higher rates of final TIMI flow grade 3 (Supplemental Materials). When the study by Desmet et al.^17^ was excluded, adenosine was also associated with a significantly higher rate of TIMI flow grade 3 (OR: 1.69; 95% CI: 1.05-2.71). Following quality assessment, 4 studies were excluded from the sensitivity analysis.^18^^,^^19^^,^^22^^,^^25^ Results remained consistent with the primary analysis (Supplemental Figures 27 to 31), with adenosine again showing a significant association with improved final TIMI flow grade 3 (OR: 1.53; 95% CI: 1.12-2.10).

### Meta-regression

Meta-regression analyses did not reveal any significant confounding effects from baseline age or sex distribution, nor from the intraprocedural use of GP IIb/IIIa inhibitors or mechanical thrombectomy (Supplemental Figures 32 to 35).

## Discussion

The results of this NMA of randomized trials can be summarized as follows: 1) among the 4 tested drugs, epinephrine and verapamil significantly improved TIMI flow grade >2; 2) significant STR was consistently achieved with epinephrine and verapamil, and with adenosine only in the frequentist analysis; and 3) all 4 agents showed only nonsignificant trends toward reducing MACE and all-cause mortality (Central Illustration).

**Central Illustration:**
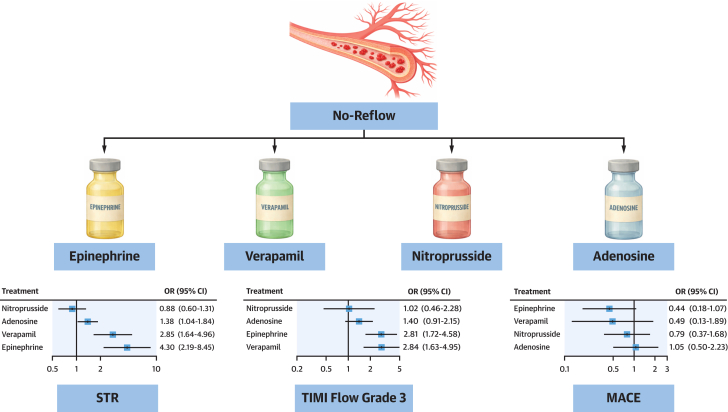
**Intracoronary Vasoactive Drugs the Treatment of No-Reflow** In primary PCI complicated by no-reflow, verapamil and epinephrine were the most effective agents for restoring coronary flow, with verapamil showing the most consistent benefit across TIMI 3 flow and STR. MACE = major adverse cardiovascular events; STR = ST-segment resolution.

Previously published meta-analyses on the subject presented heterogeneous inclusion criteria, as well as sparse treatment options and timing. Laborante et al^32^ only included studies comparing intracoronary or intravenous adenosine vs placebo (increasing heterogeneity), whereas Niu et al^33^ assessed the effect of 7 intracoronary agents (adenosine, anisodamine, diltiazem, nicorandil, nitroprusside, urapidil, and verapamil), most of which are not used in daily practice, and did not include epinephrine.

Methodologically, our meta-analysis specifically focused on 4 routinely-used drugs for the treatment of the no-flow phenomenon (adenosine, epinephrine, nitroprusside, and verapamil), with intracoronary administration through a microcatheter or an over-the-wire balloon. We excluded studies combining the 4 pharmacological strategies together and only allowed combination with nonvasoactive agents such as GP IIb/IIIa inhibitors. Furthermore, we only included randomized controlled studies. Such strict criteria for study selection enabled a more reliable analysis of each single drug separately. Thus, all the included studies could be comparable, as it can be appreciated by the low heterogeneity and by the “leave-one” analysis that assessed the influence of each study on the overall extracted results.

Classically, the management of no-reflow depends on intracoronary vasodilation. Overall, in our NMA only epinephrine and verapamil showed a statistically significant improvement in flow restoration (both defined as TIMI flow grade 3 and TIMI flow grade >2) as well as in STR. Although these 2 agents share a common final effect of vasodilatation (epinephrine through beta 2 receptors, verapamil through calcium channel blockage), epinephrine also features beta 1 receptor-mediated inotropic and chronotropic stimulation, which can increase the coronary flow. Previous studies showed that intracoronary epinephrine administration for the management of no-flow during primary PCI yielded significantly better coronary flow patterns compared to those treated with conventional agents such as nitrates, adenosine, thrombectomy, and Gp IIb/IIIa inhibitors.^34^ As far as the 2 remaining agents, both are regarded as effective for reducing the incidence of no-reflow; however, adenosine can increase the risk of atrioventricular block and has a relatively short half-life, which requires repeated administrations.^35^ Moreover, both adenosine and nitroprusside increase the risk dose-related hypotension.^36^ The findings of this NMA are somewhat in contrast with the current practice, where adenosine is the most widely used drug as a first line treatment; epinephrine is commonly a second line drug after failure of flow restoration with adenosine, and, on the other hand, verapamil is rarely used.

Although angiographic (ie, TIMI flow grade) or instrumental (ie, STR) benefits were found in our NMA for some agents (epinephrine and verapamil), no significant benefit was found regarding clinical outcomes. This could have several explanations: first, TIMI flow grade and STR bear the limitations of all surrogate endpoints, which do not fully capture the complexity of clinical and late-manifesting outcomes.^37^ Second, the no-reflow phenomenon is a transient phenomenon, which can be acutely and angiographically solved by the vasoactive agent, without, nevertheless, yielding a long-term resolution of the underlying myocardial damage. Third, the studies assessed the clinical outcomes with relatively short follow-up periods (maximum of 6 months) and this could miss significant differences in the long-term. Fourth, the majority of the included studies presented relatively low sample sizes (on average between 100 and 200 patients) and could therefore be underpowered for detecting clinical outcomes such as MACE or death. Fifth, the exact mechanism behind the no-flow/no-reflow phenomenon is still debated and multifactorial, with possible concomitant implications of microvascular obstruction by debris, endothelial dysfunction as well as reperfusion injury^30^: it is possible that each tested drug targets some, but not all, of these mechanisms. And finally, the effects of the tested agents could be confounded by underlying and pre-existing cardioprotective therapies that vary from patient to patient (such as antiplatelet therapy, beta-blockers, and statins).

### Study limitations

The findings of this NMA should be interpreted considering some limitations. First, we acknowledge that the broad definition of no-reflow used in the included studies, even if reflecting the terminology adopted in the original study, may introduce conceptual heterogeneity and potential classification bias. Then, the included studies all compared the 4 tested drugs in a different fashion, such as one-to-one comparison of a drug with another, or with a placebo, or a comparison of 3 different strategies; moreover, no direct comparison between epinephrine and nitroprusside was present. However, as already mentioned, the absence of studies where the different strategies were combined provide a clear separation of the pharmacological effects from which our findings can be inferred. Then, some procedural differences were present, such as the timing and dosage of the drugs among studies and the usage, at operator discretion, of balloon inflation before intracoronary infusion, potentially affecting our results. This aspect mainly reflects the absence of a shared consensus on the best approach to the reflow phenomenon, procedurally and pharmacologically; in this context, our NMA could guide future studies to clear such gaps in evidence. As already mentioned in the discussion section, the relatively small sample sizes and short follow-up also represent limitations that underpower each single study for the analysis of clinical outcomes. Nevertheless, due to the relatively low number of studies, the meta-regression analysis may lack sufficient statistical power and therefore results are exploratory and should not be considered definitive. Moreover, due to the small number of studies contributing to each comparison, funnel plots offer only limited interpretability and may not reliably detect publication bias; therefore, these assessments should be viewed as exploratory.

## Conclusions

In primary PCI complicated by no-reflow, verapamil and epinephrine were associated with significantly higher odds of achieving final TIMI flow grade 3 compared with controls, with verapamil showing the most consistent benefit across TIMI flow grade 3 and STR. Adenosine and nitroprusside showed no clear association compared with controls. Further studies are needed to clarify optimal agent selection, dosing, and timing of administration.

## Funding support and author disclosures

The Department of Cardiology of the Leiden University Medical Center received unrestricted research grants from 10.13039/100011949Abbott Vascular, 10.13039/100004326Bayer, 10.13039/501100005035Biotronik, 10.13039/100008497Boston Scientific, 10.13039/100006520Edwards Lifesciences, 10.13039/100006775GE Healthcare, and 10.13039/100004374Medtronic. Dr Montero-Cabezas received a research grant from Shockwave Medical and speaker fees from 10.13039/100020297Abiomed, 10.13039/100008497Boston Scientific, and 10.13039/100020501Penumbra Inc. Dr De Luca received speaker fees from 10.13039/100002429Amgen, 10.13039/501100015704Aspen, 10.13039/100004325AstraZeneca, 10.13039/100004326Bayer, 10.13039/100001003Boehringer Ingelheim, Chiesi, Daiichi Sankyo, Eli Lilly, Menarini, Novonordisk, Pfizer/Bristol-Myers Squibb, Sanofi, and Servier. All other authors have reported that they have no relationships relevant to the contents of this paper to disclose.
