# Supplementary material for: Intracoronary Vasoactive Therapy for No-Reflow During Primary PCI: A Network Meta-Analysis of Randomized Trials
Source: JACC Adv. 2026 Feb 25;5(3):102599. doi: 10.1016/j.jacadv.2026.102599 (PMC12955103; doi:10.1016/j.jacadv.2026.102599)

**
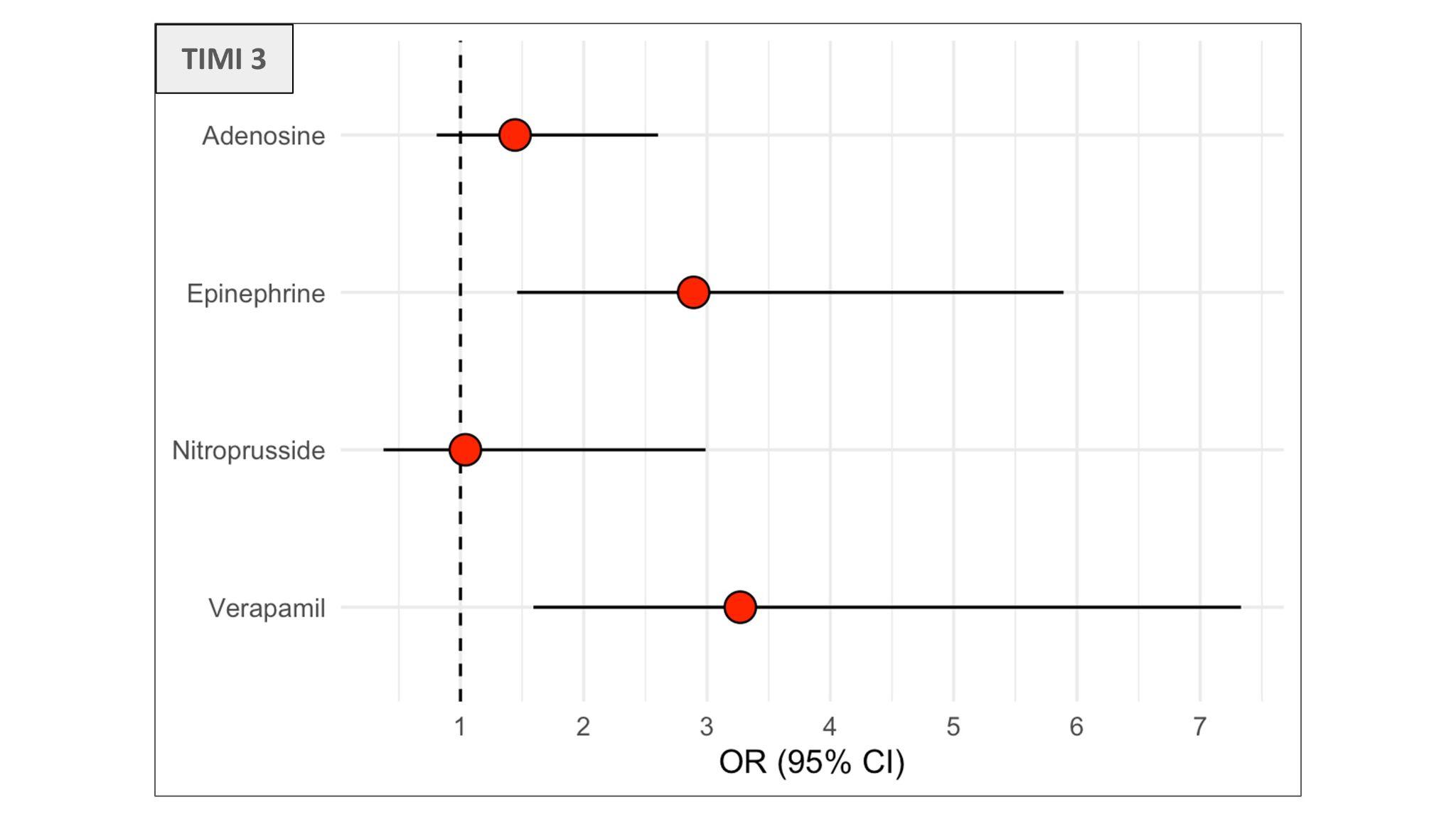
**

**Figure S1.** Bayesian forest plot of the odds ratios (ORs) and 95% credible intervals for achieving TIMI 3 flow with different intracoronary medications.

**
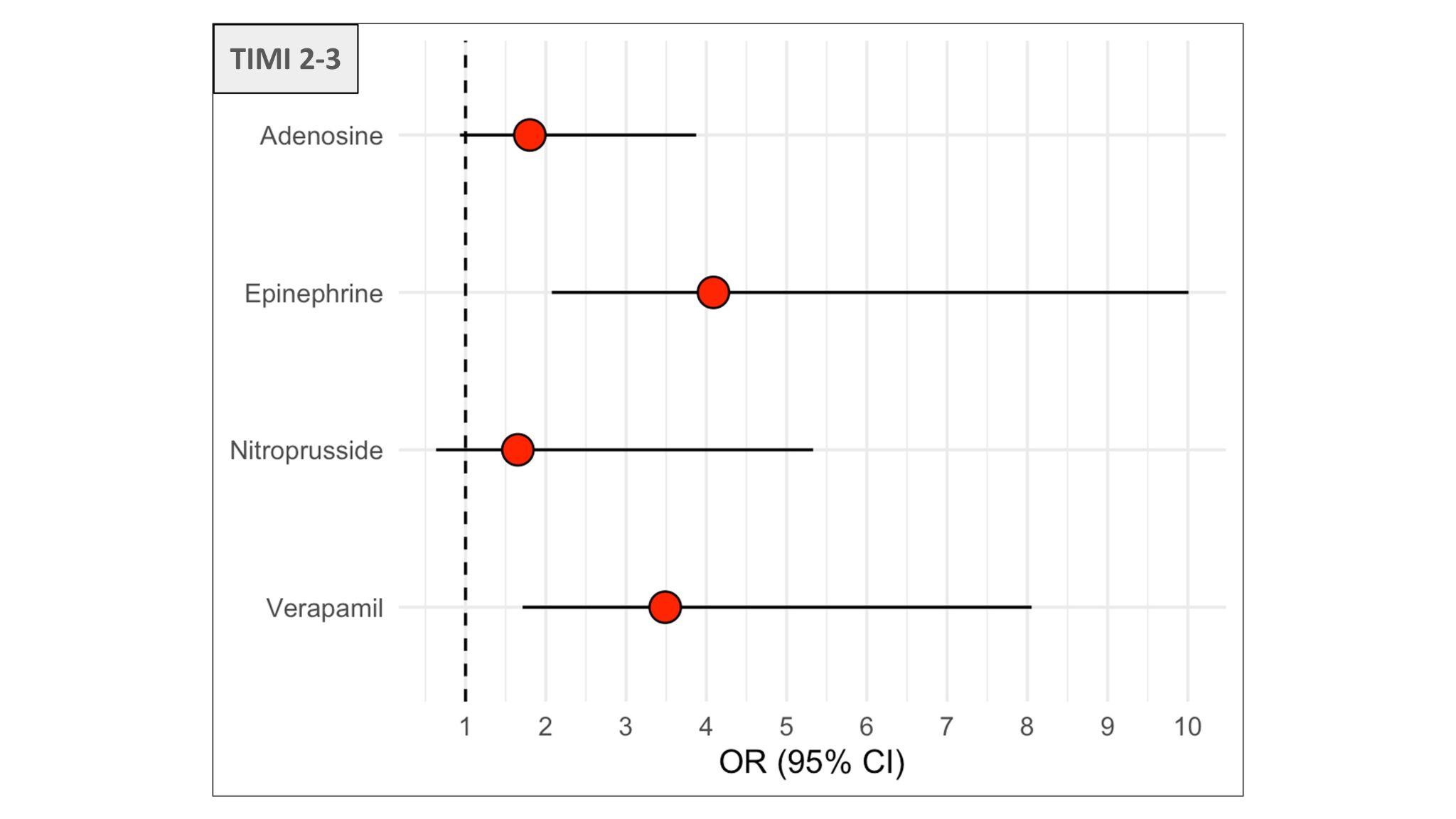
Figure S2.** Bayesian forest plot of the odds ratios (ORs) and 95% credible intervals for achieving TIMI 2-3 flow with different intracoronary medications.

**
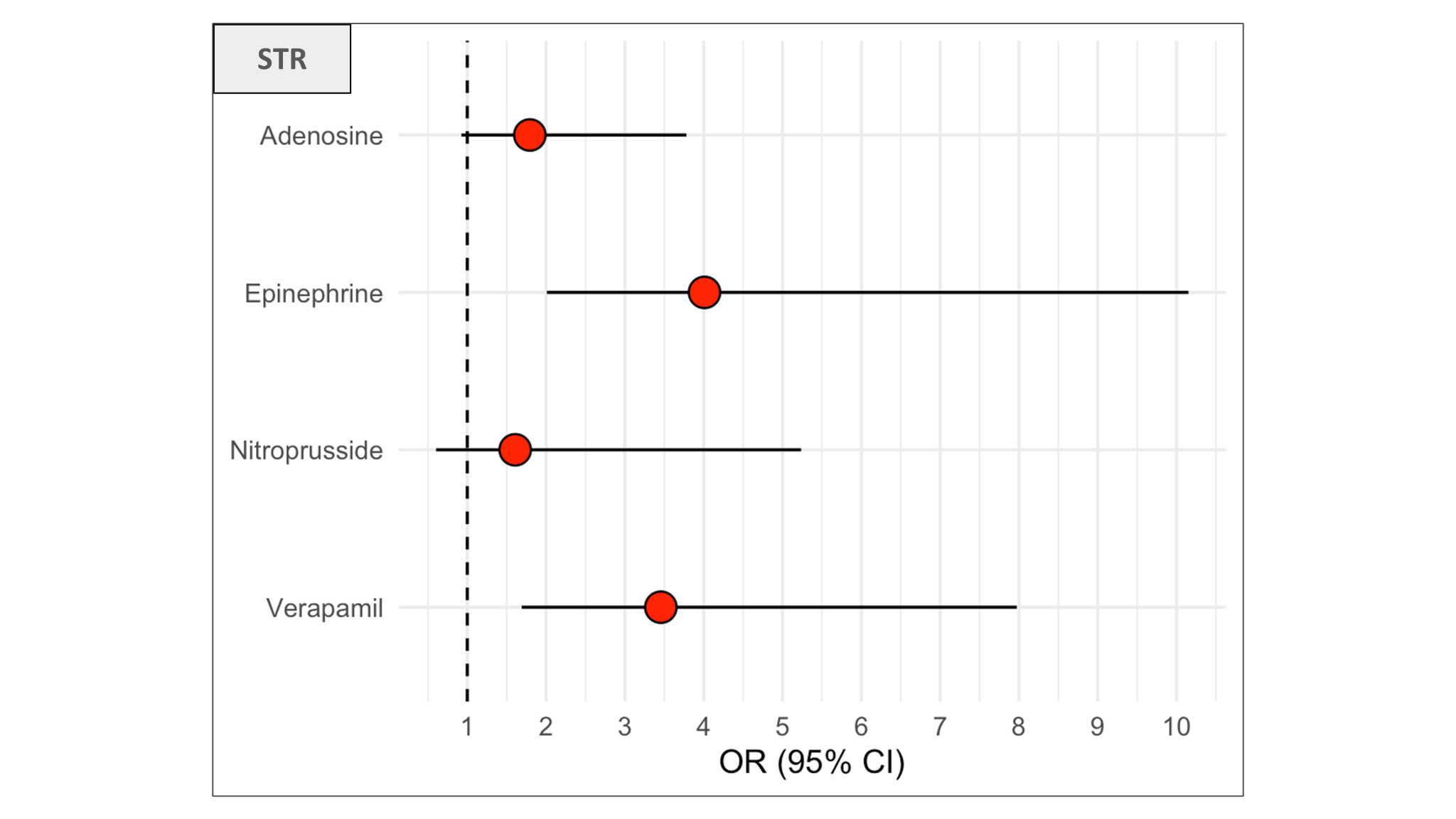
Figure S3.** Bayesian forest plot of the odds ratios (ORs) and 95% credible intervals for achieving STR with different intracoronary medications. *STR: ST-tract elevation resolution.*

**
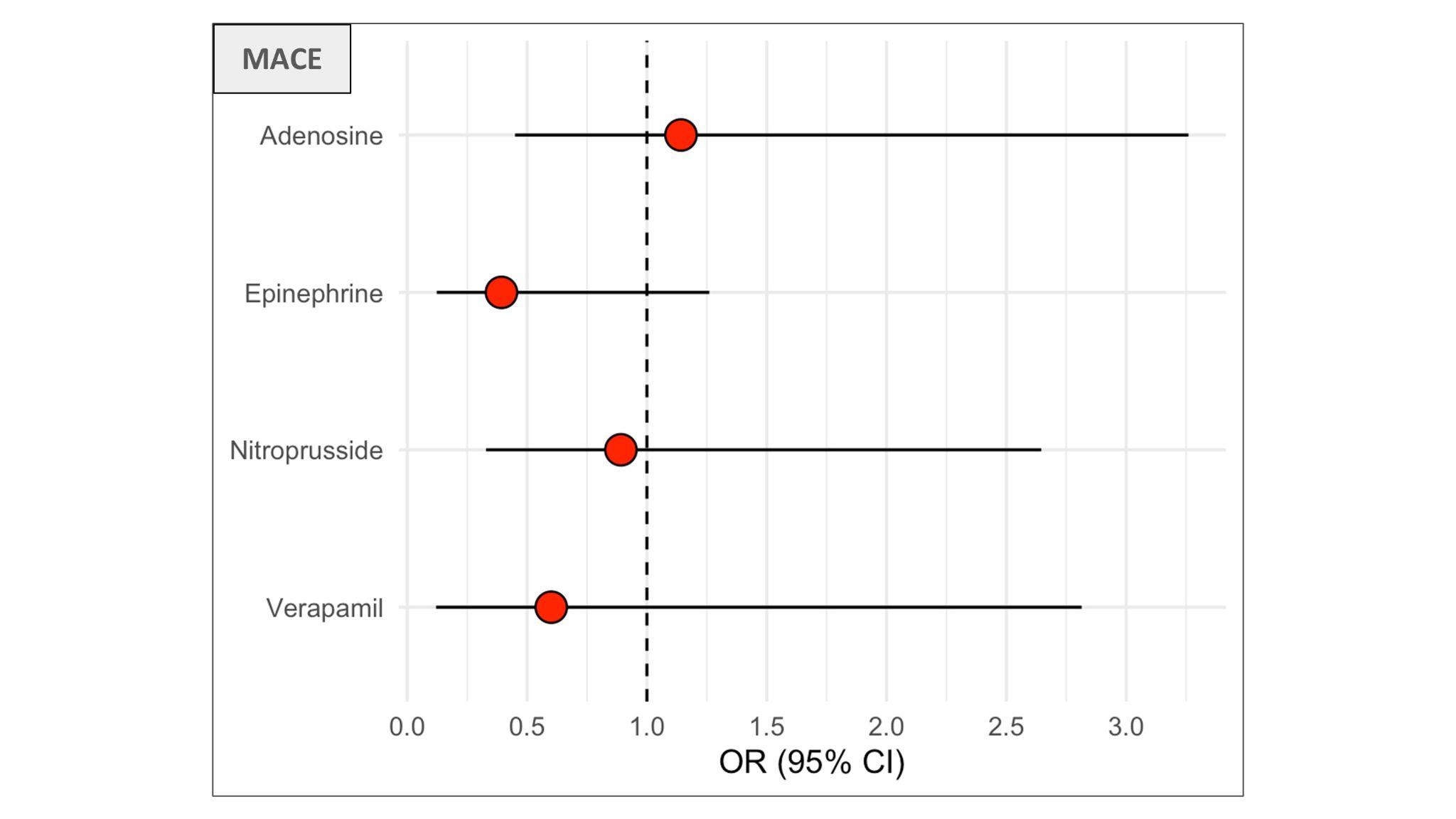
Figure S4.** Bayesian forest plot of the odds ratios (ORs) and 95% credible intervals for achieving MACE with different intracoronary medications.

*
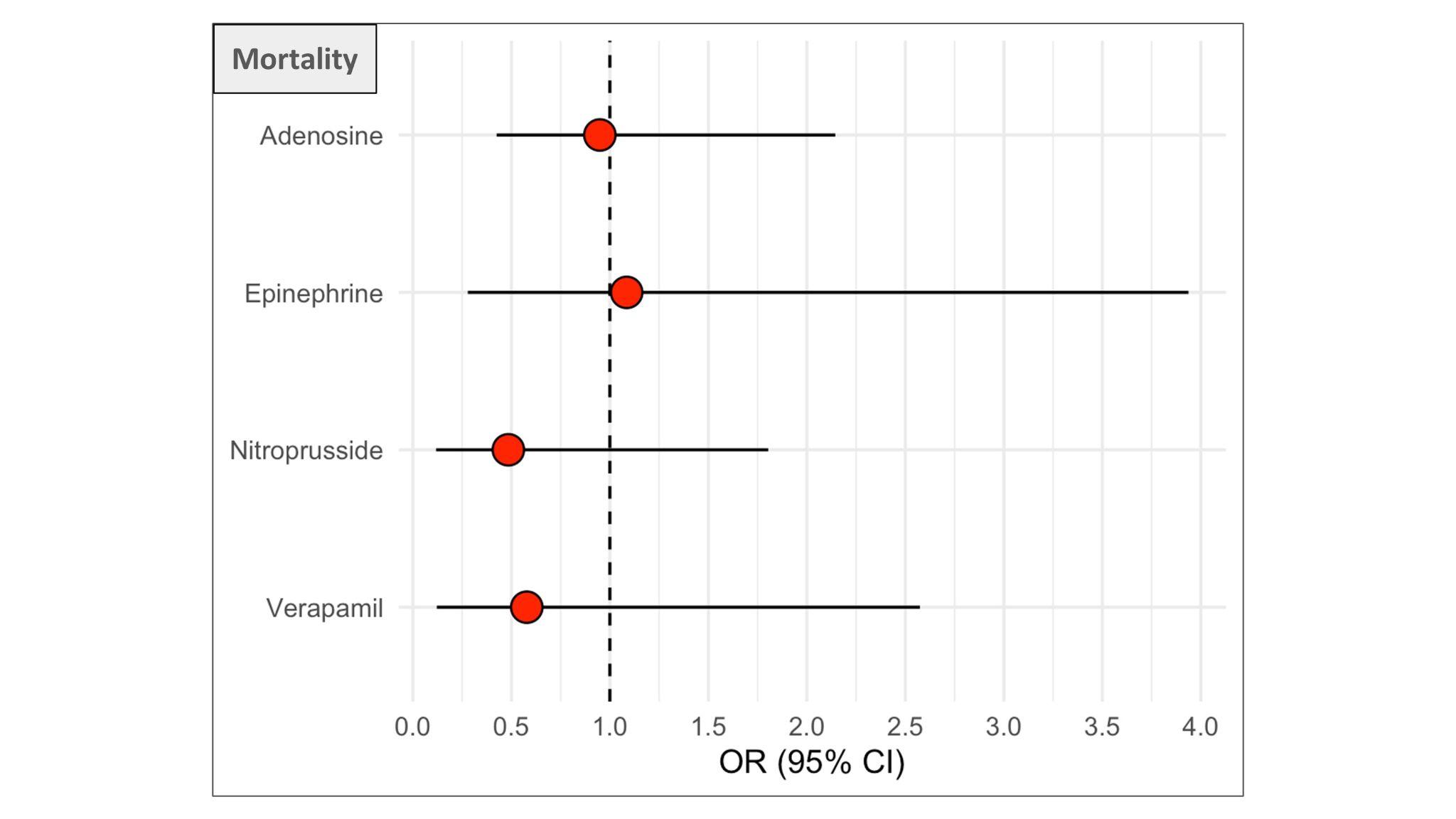
*

**Figure S5.** Bayesian forest plot of the odds ratios (ORs) and 95% credible intervals for achieving mortality with different intracoronary medications.

**
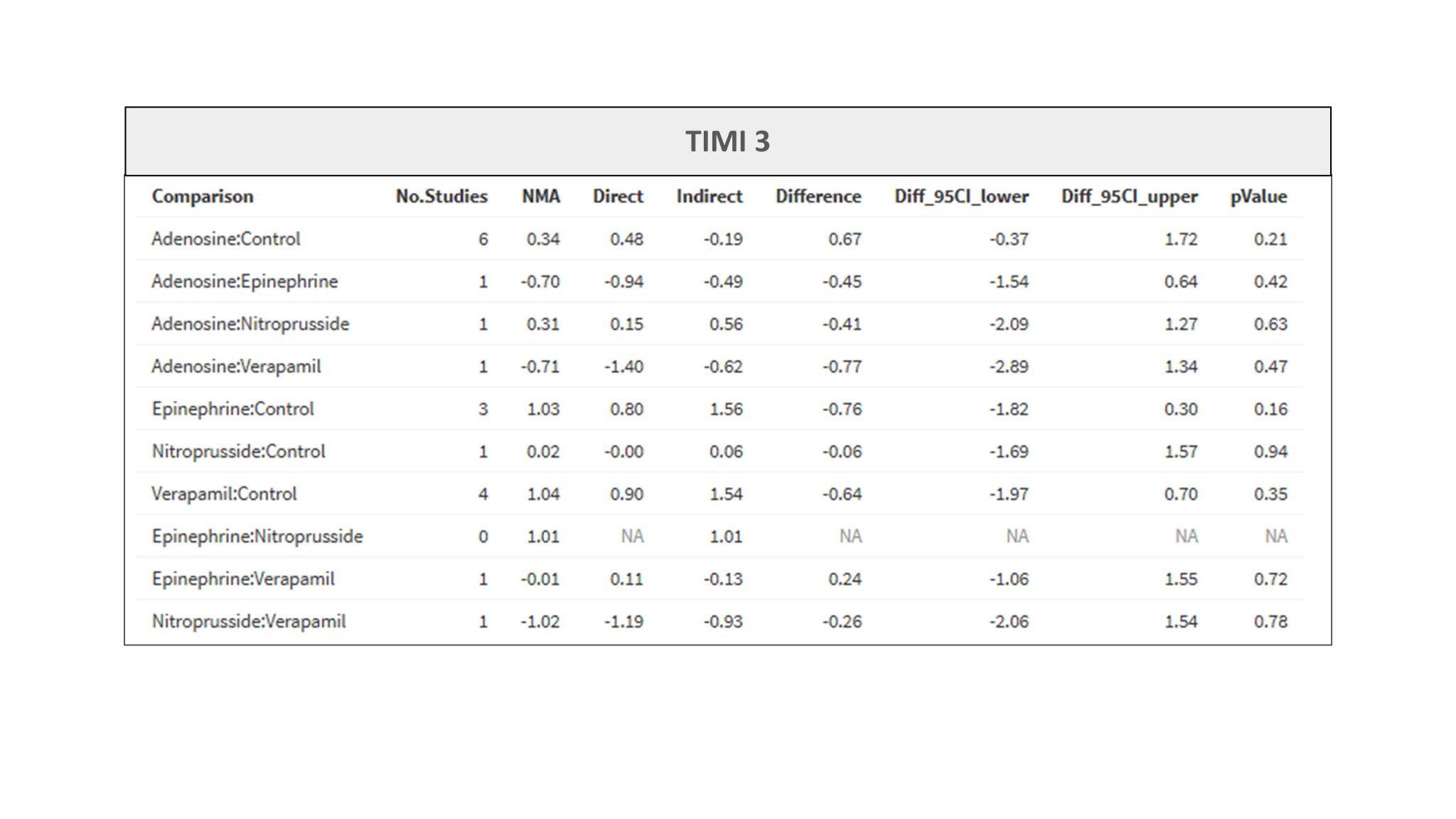
**

**Figure S6.** Inconsistency table showing discrepancies between direct and indirect comparisons in the frequentist analysis for achieving TIMI 3 flow with different intracoronary medications.

**
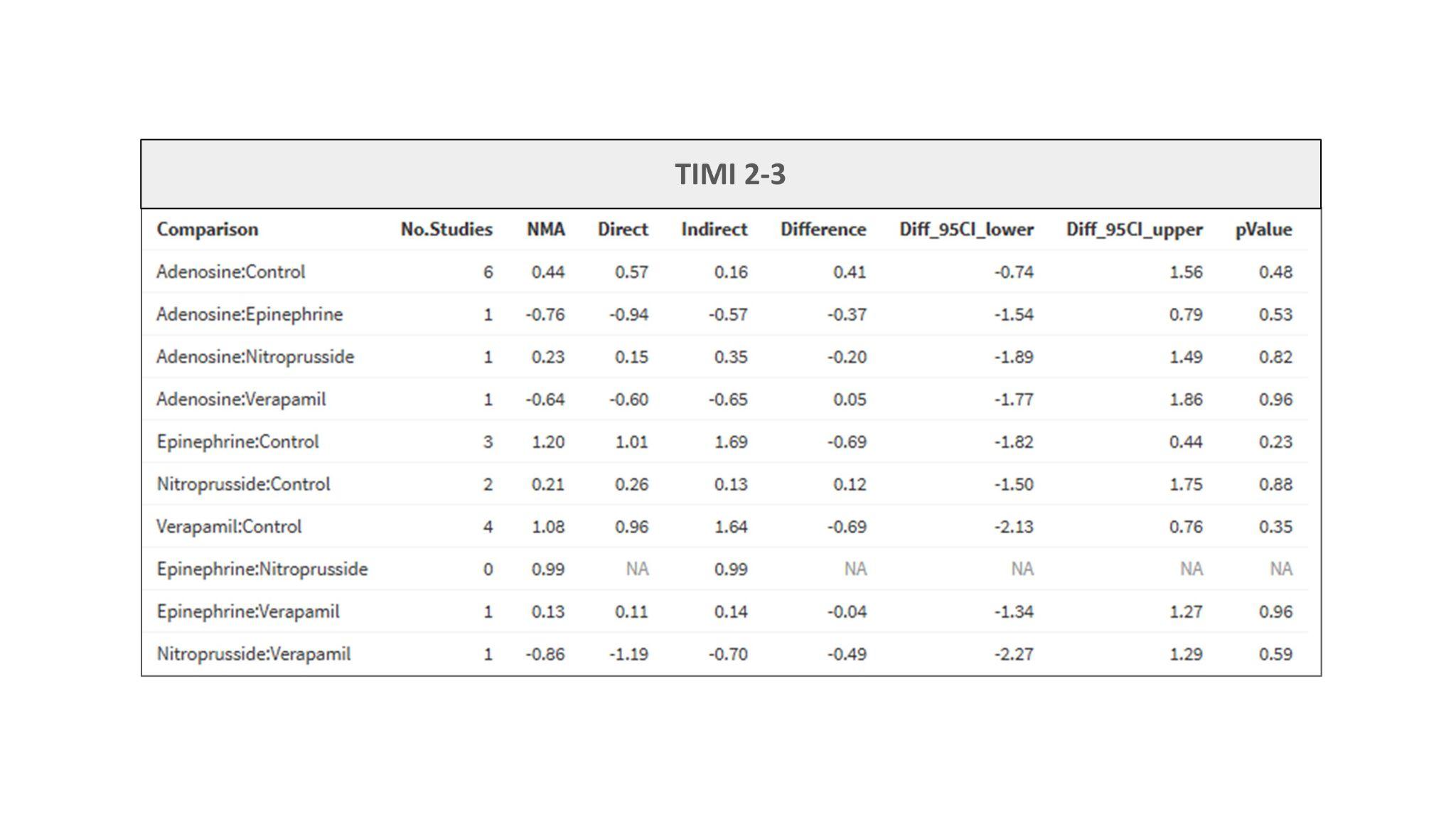
Figure S7.** Inconsistency table showing discrepancies between direct and indirect comparisons in the frequentist analysis for achieving TIMI 2-3 flow with different intracoronary medications.

**
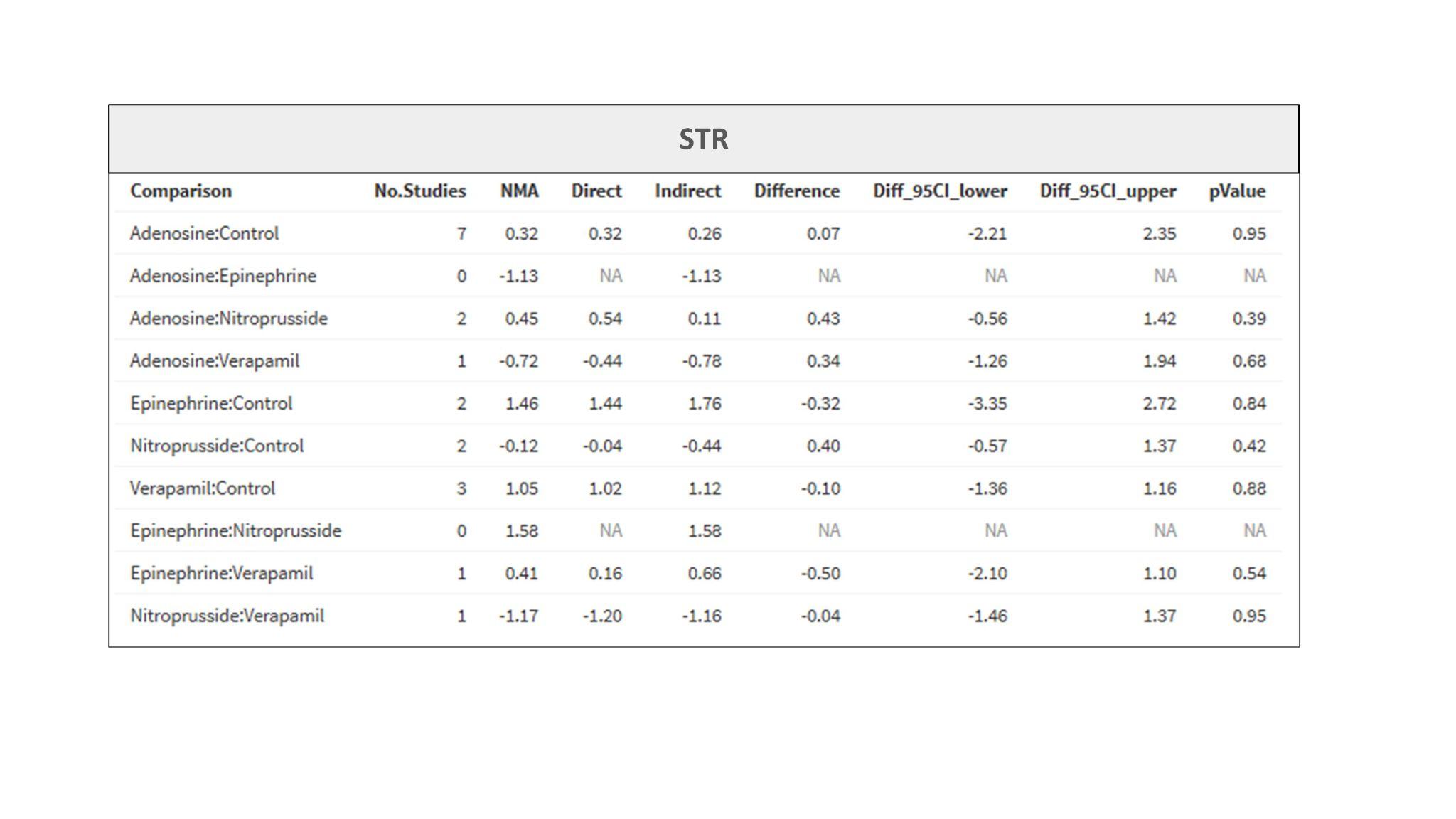
**

**Figure S8.** Inconsistency table showing discrepancies between direct and indirect comparisons in the frequentist analysis for STR with different intracoronary medications.

**
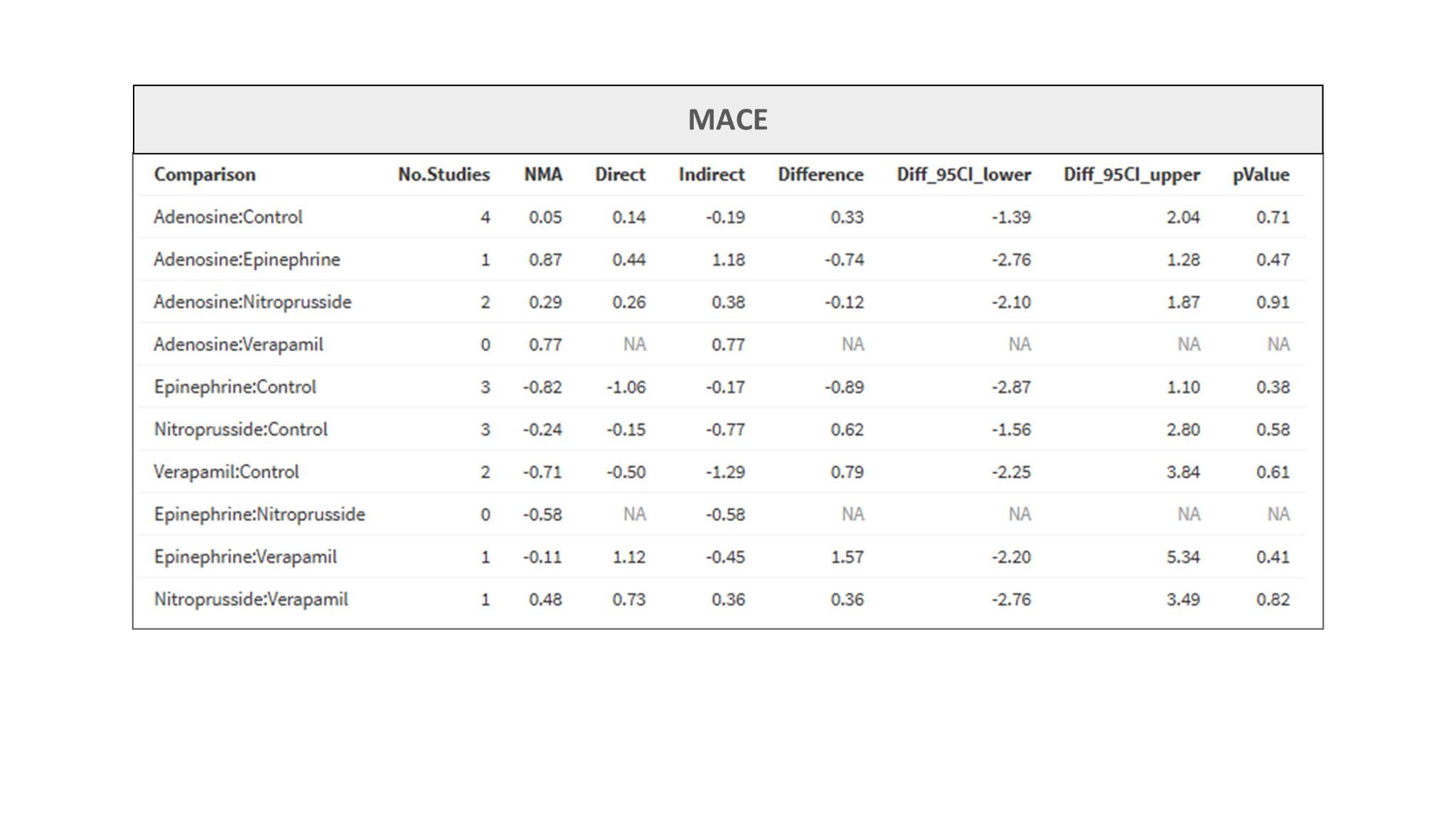
Figure S9.** Inconsistency table showing discrepancies between direct and indirect comparisons in the frequentist analysis for MACE with different intracoronary medications.

**
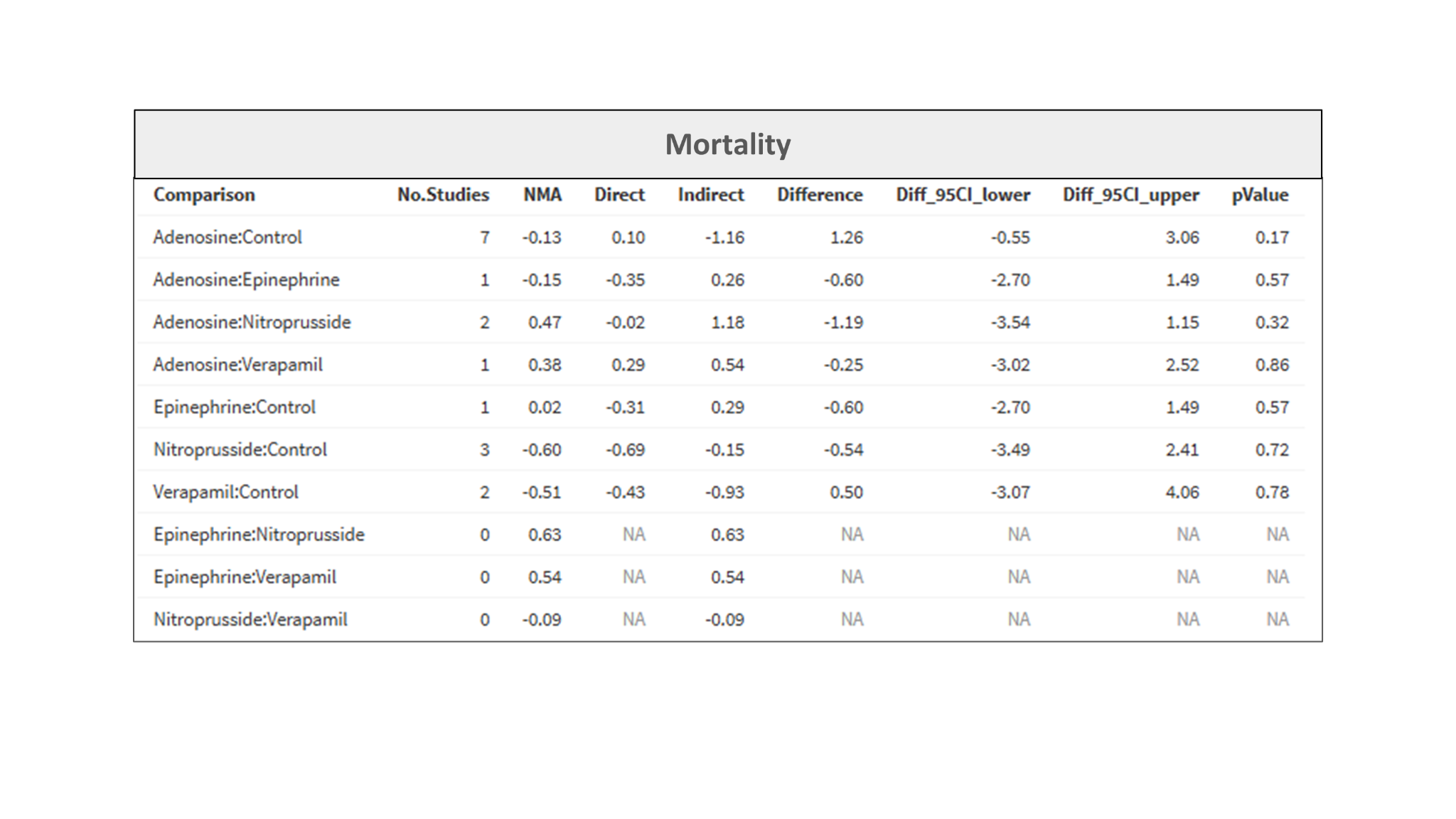
**

**Figure S10.** Inconsistency table showing discrepancies between direct and indirect comparisons in the frequentist analysis for mortality with different intracoronary medications.

**
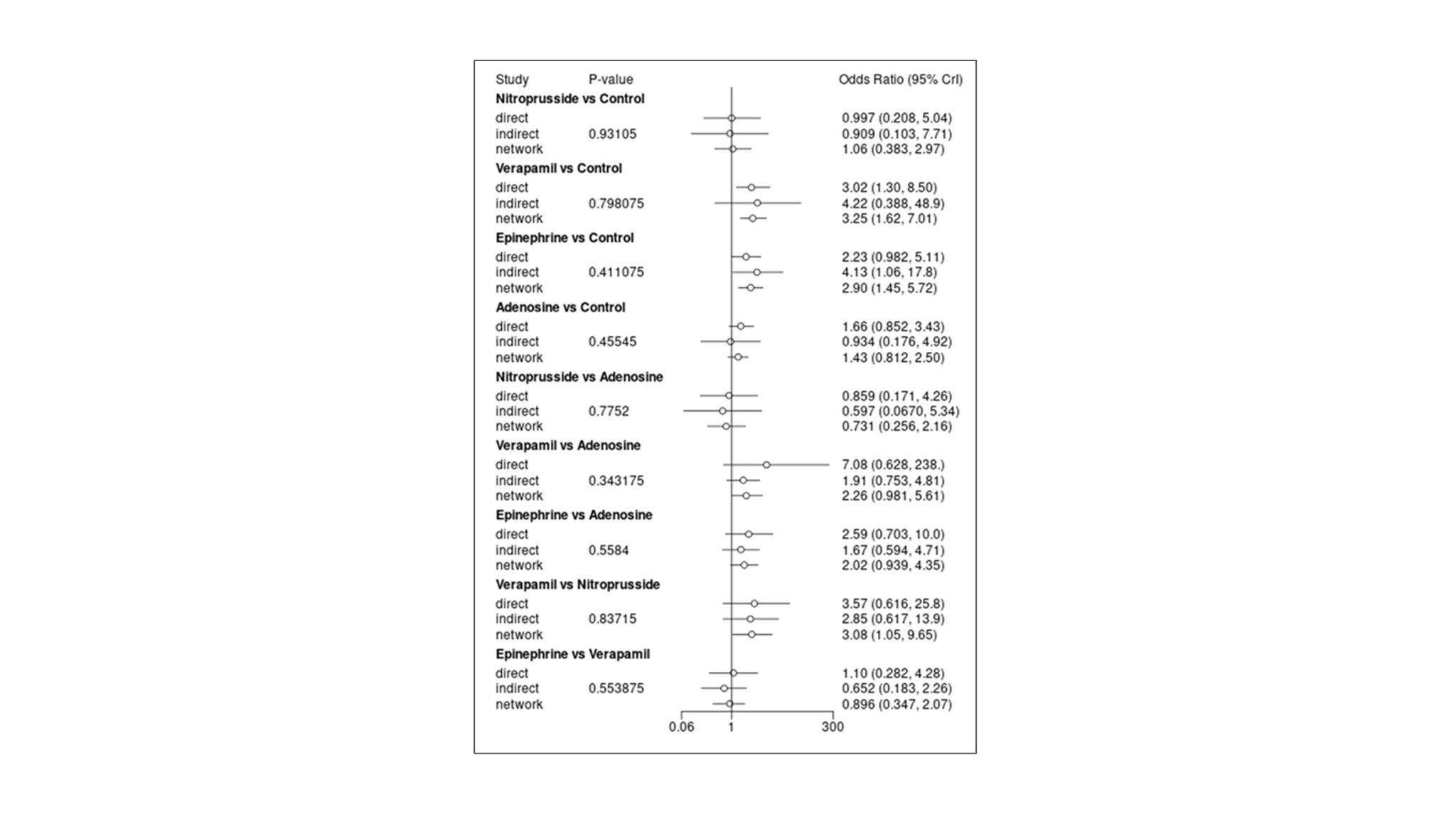

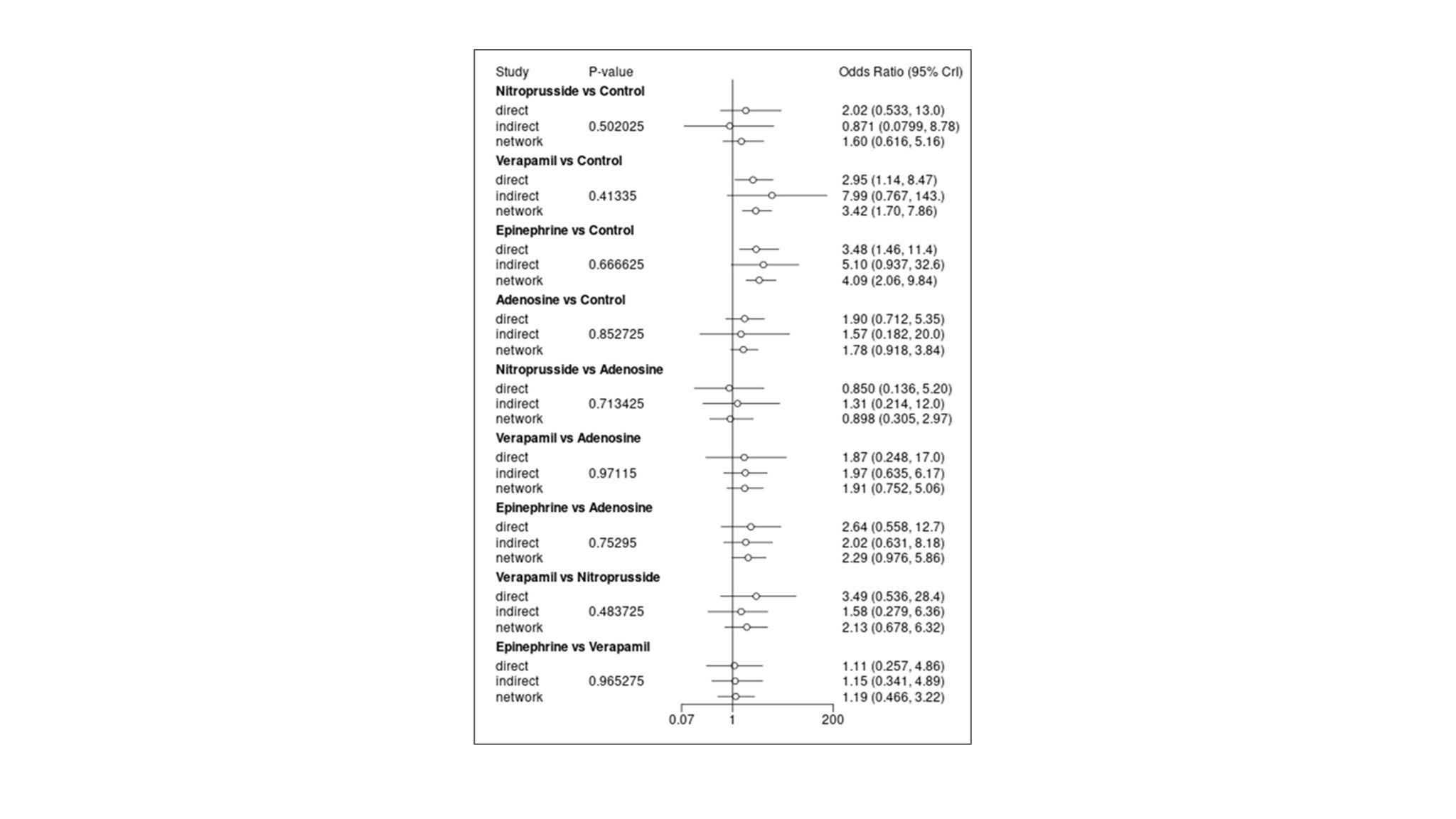
**

**Figure S11.** Comparative inconsistency assessment in the Bayesian network meta-analysis. The left panel refers TIMI 3 flow and TIMI 2–3 flow.


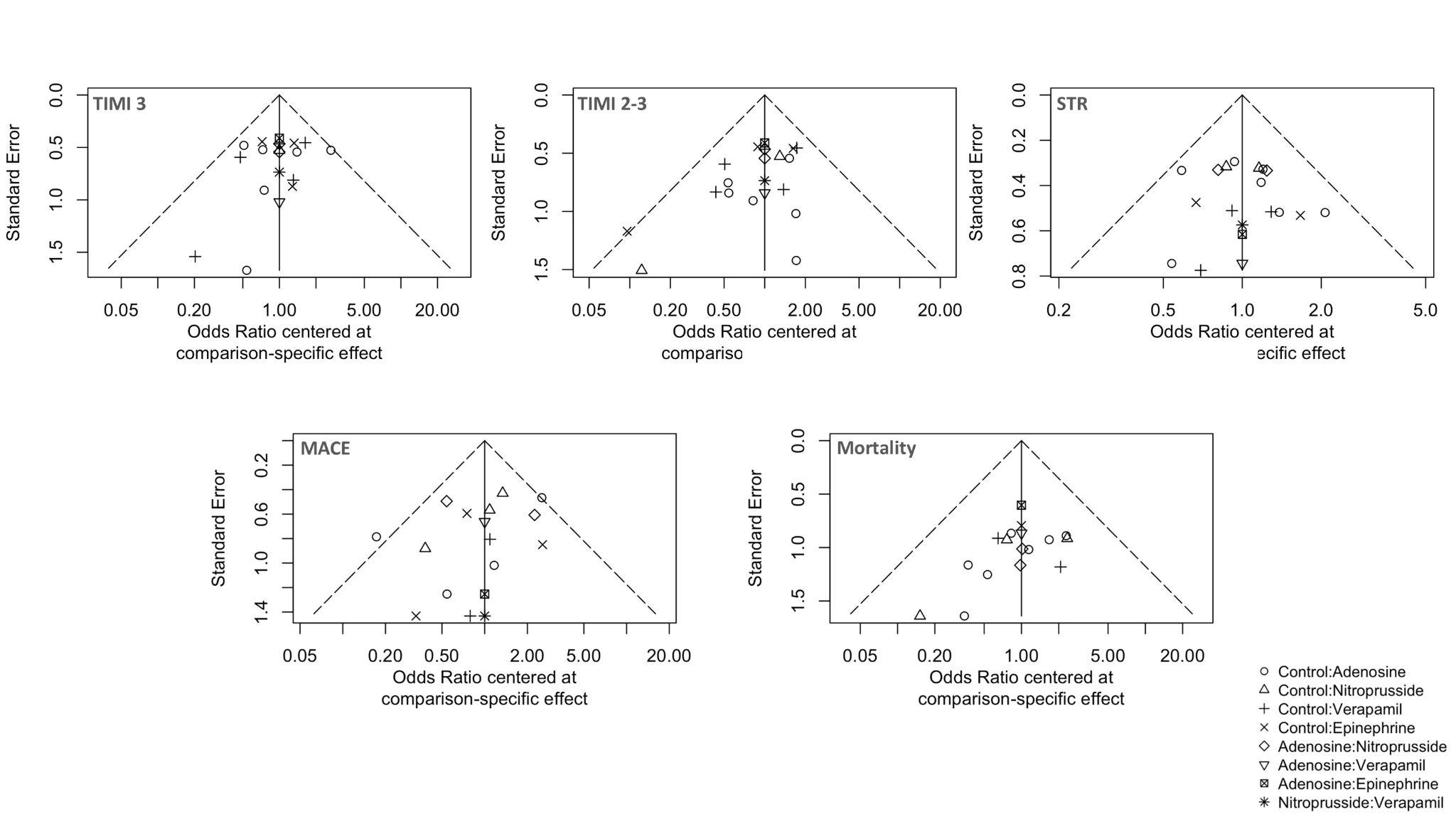


**Figure S12.** Funnel plots across TIMI 3, TIMI 2–3 flow, STR, MACE, and mortality outcomes.

**
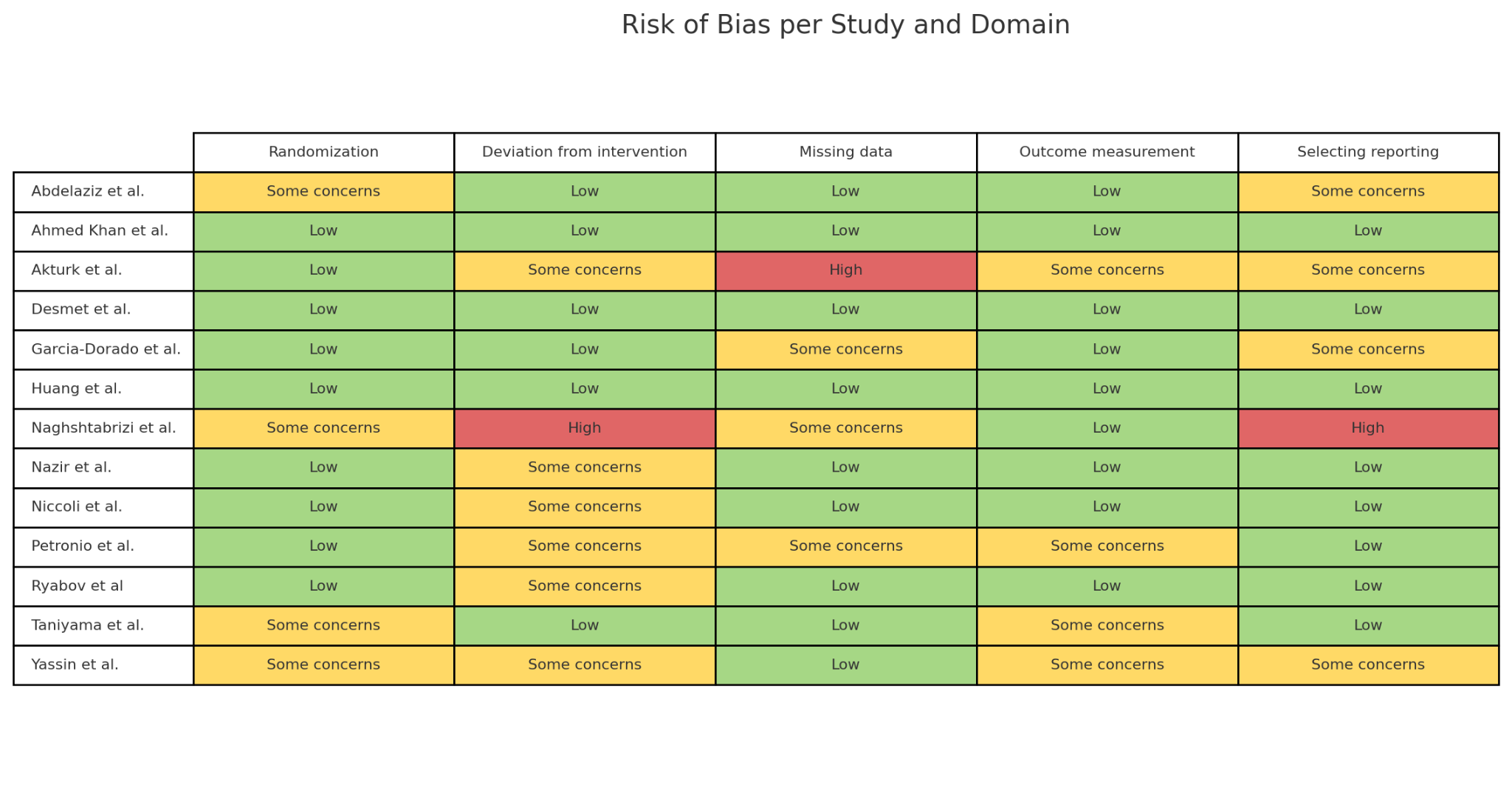
**

**Figure S13.** Risk of Bias 2 (RoB 2) assessment across included randomized controlled trials.

**
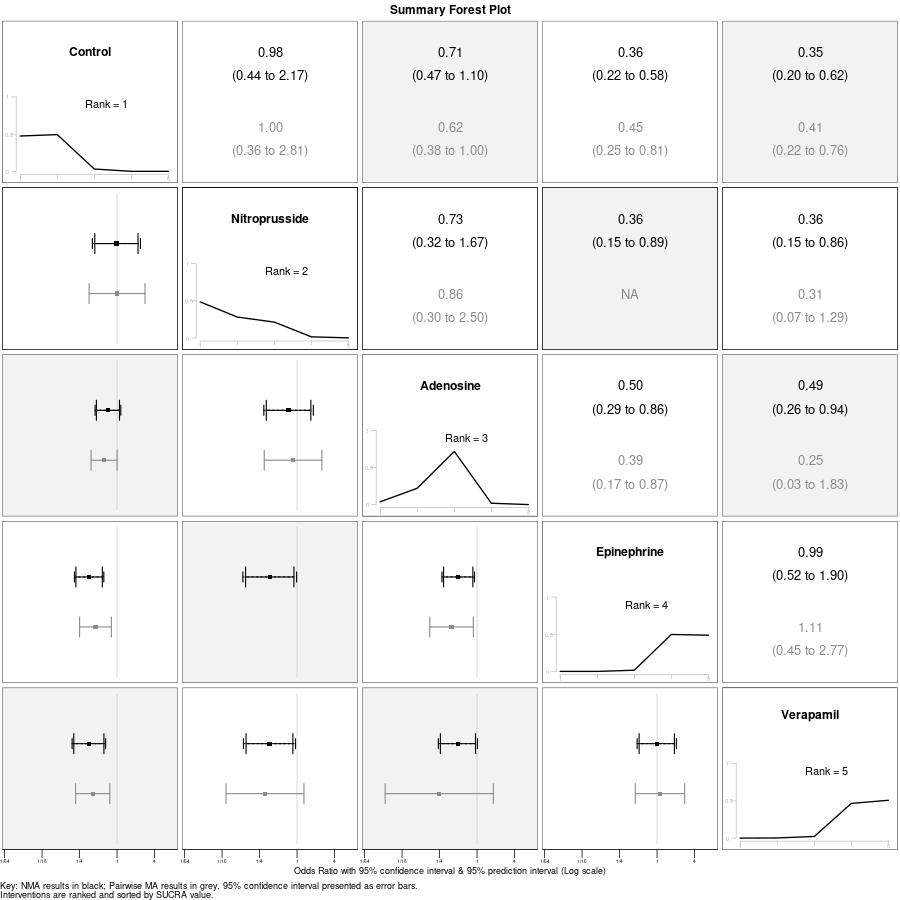
**

**Figure S14.** League Table Presenting Pairwise Comparisons of Pharmacologic Treatments for final TIMI 3 flow, Including Odds Ratios with 95% Confidence and Prediction Intervals, and SUCRA-Based Ranking.


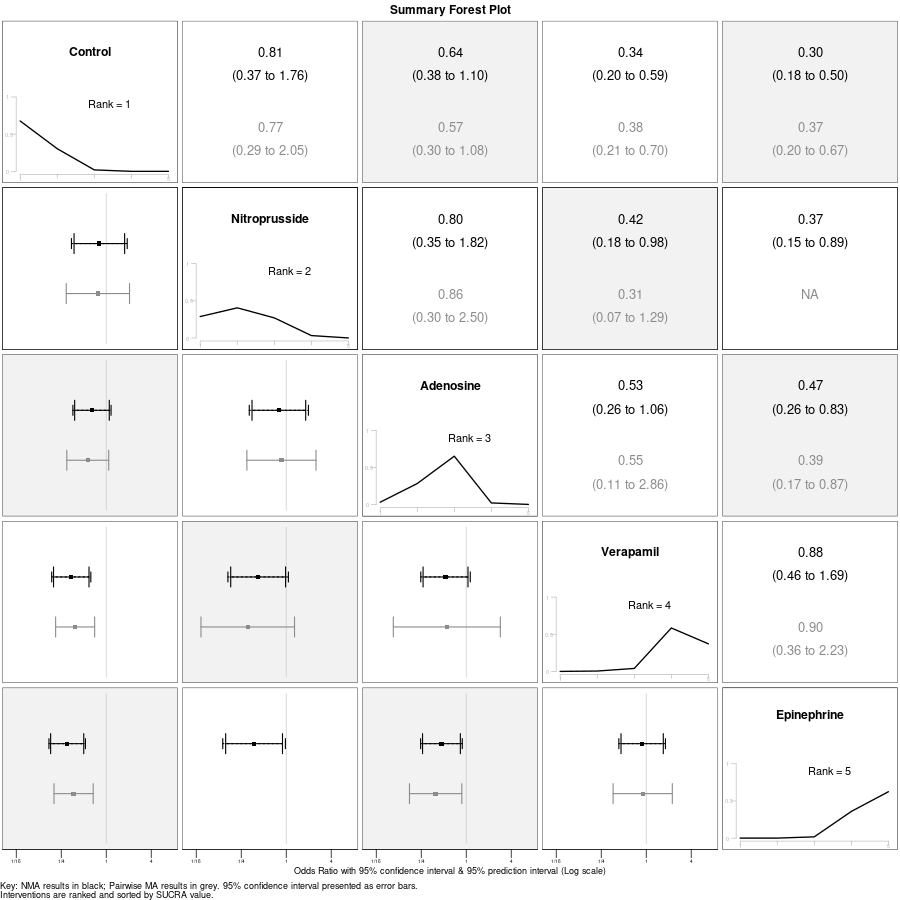


**Figure S15.** League Table Presenting Pairwise Comparisons of Pharmacologic Treatments for final TIMI 2-3 flow, Including Odds Ratios with 95% Confidence and Prediction Intervals, and SUCRA-Based Ranking.


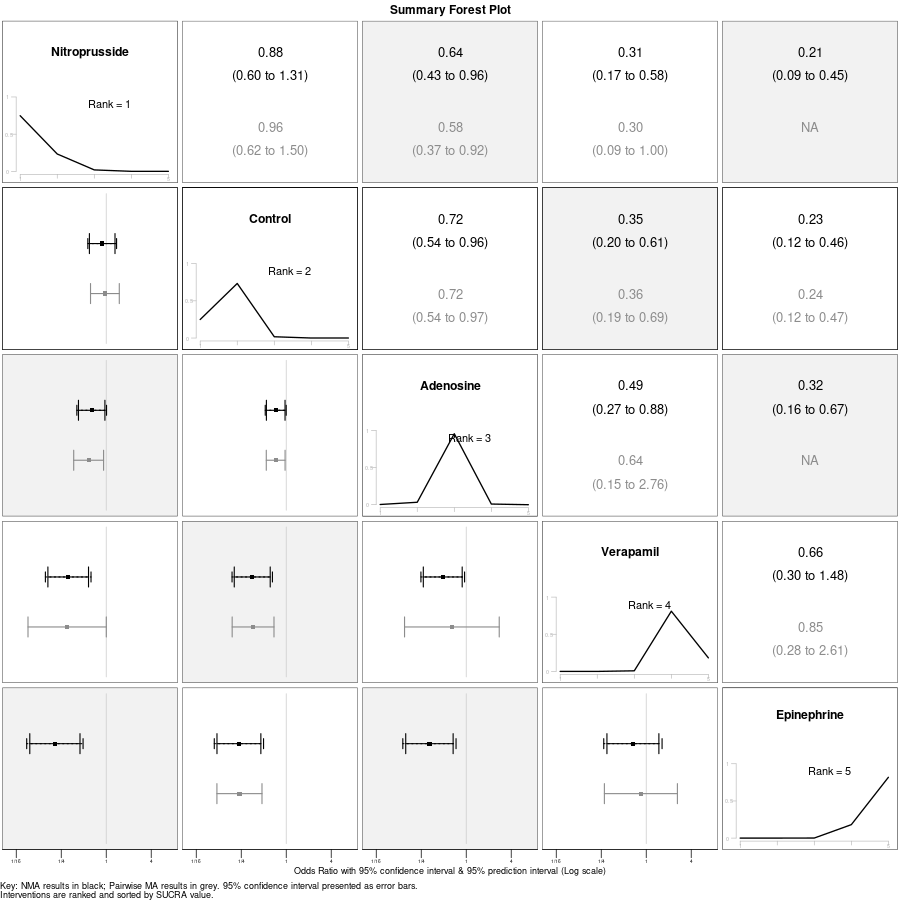


**Figure S16.** League Table Presenting Pairwise Comparisons of Pharmacologic Treatments for STR, Including Odds Ratios with 95% Confidence and Prediction Intervals, and SUCRA-Based Ranking.


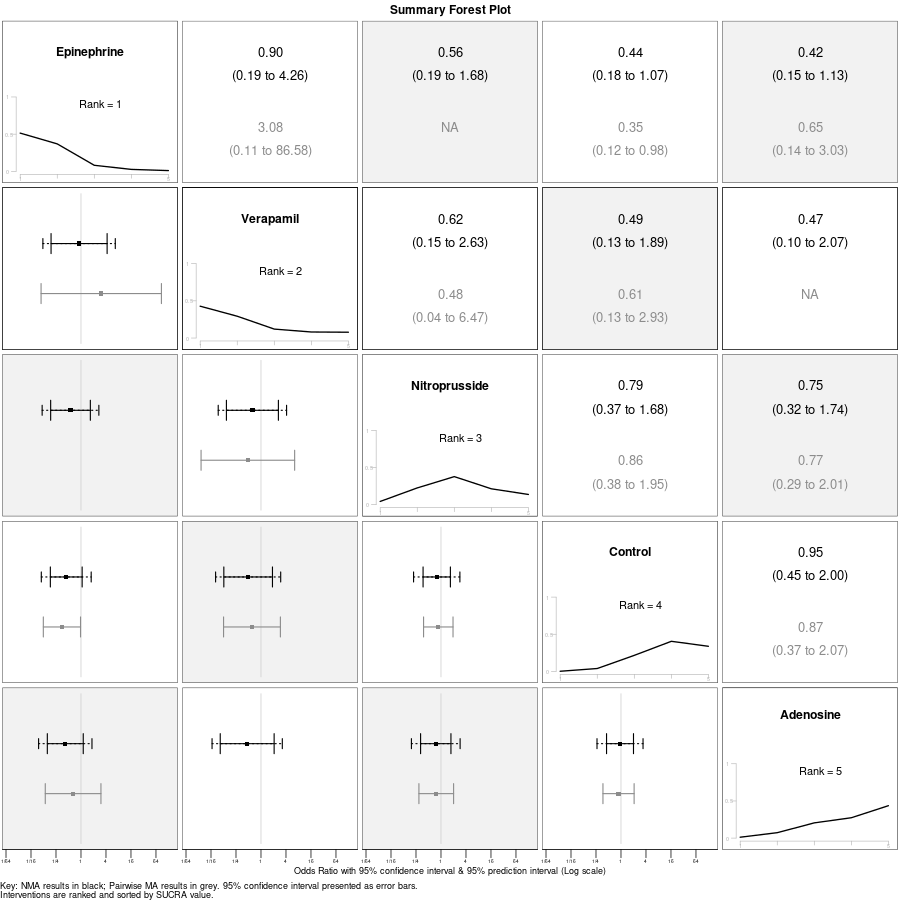


**Figure S17.** League Table Presenting Pairwise Comparisons of Pharmacologic Treatments for MACE, Including Odds Ratios with 95% Confidence and Prediction Intervals, and SUCRA-Based Ranking.


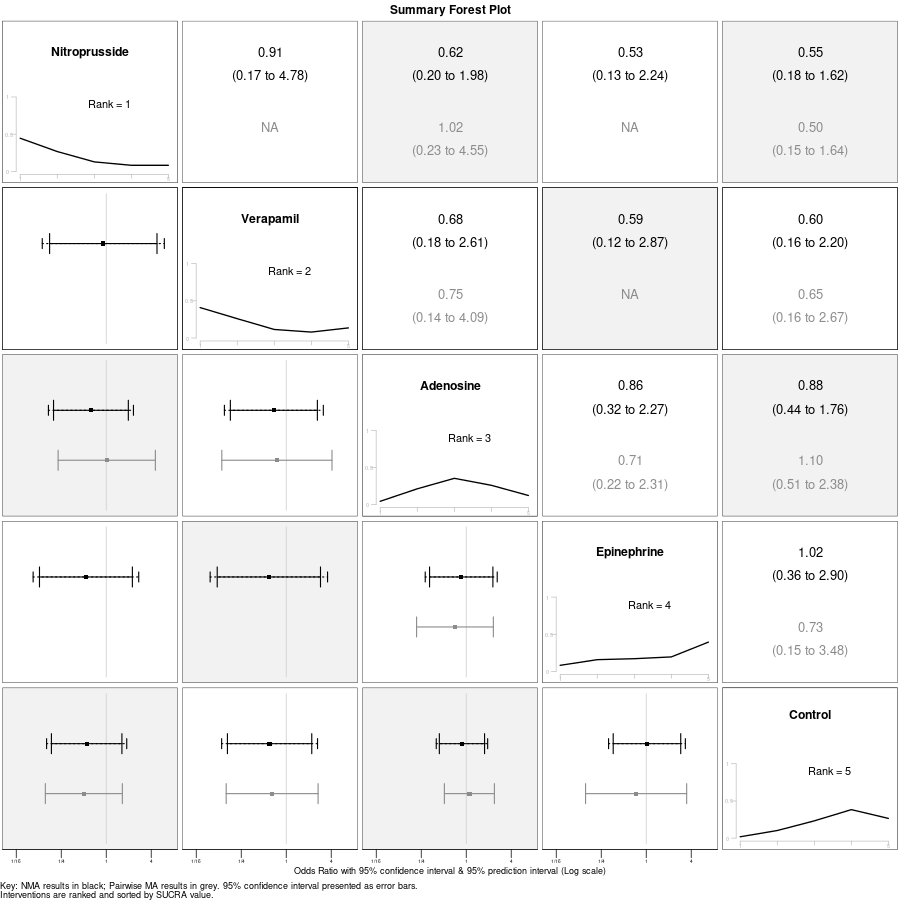


**Figure S18.** League Table Presenting Pairwise Comparisons of Pharmacologic Treatments for mortality, Including Odds Ratios with 95% Confidence and Prediction Intervals, and SUCRA-Based Ranking.

**
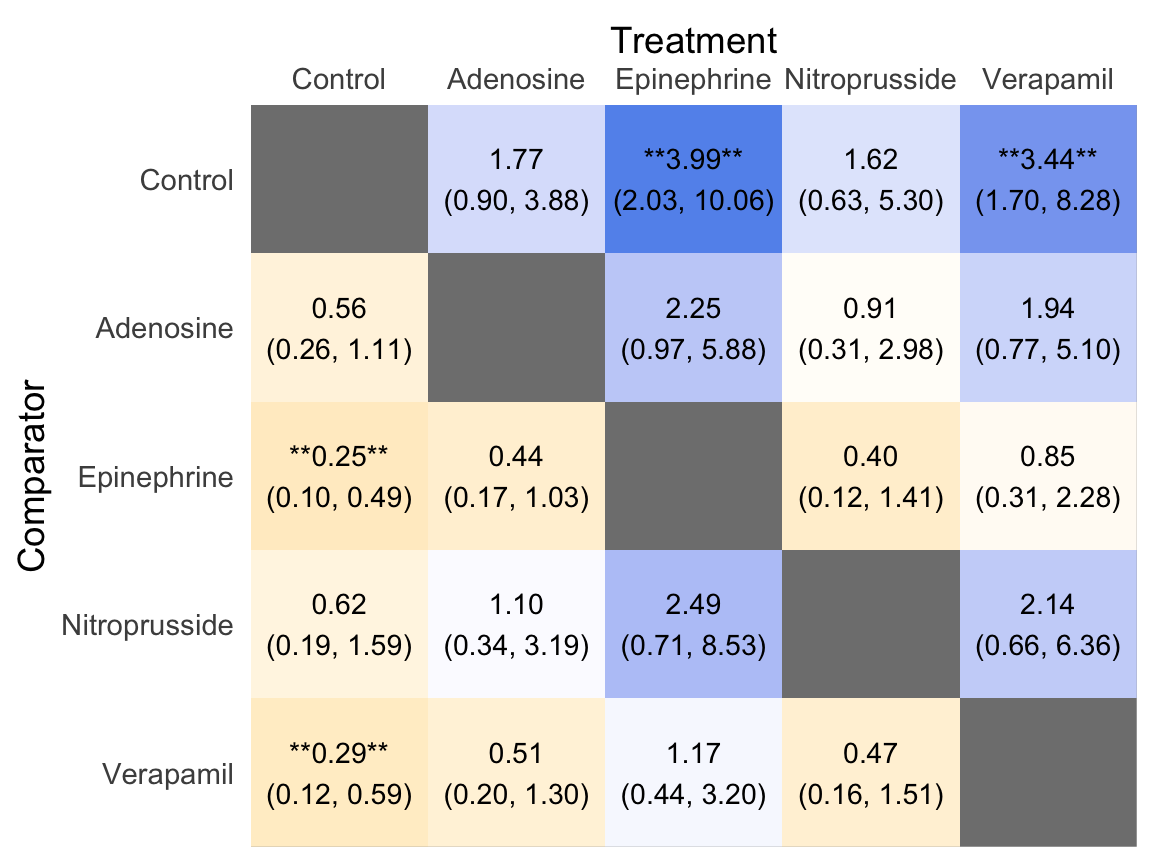
**

**Figure S19.** Bayesian Heatmap Showing Pairwise Odds Ratios for Achieving TIMI 2-3 Flow

**
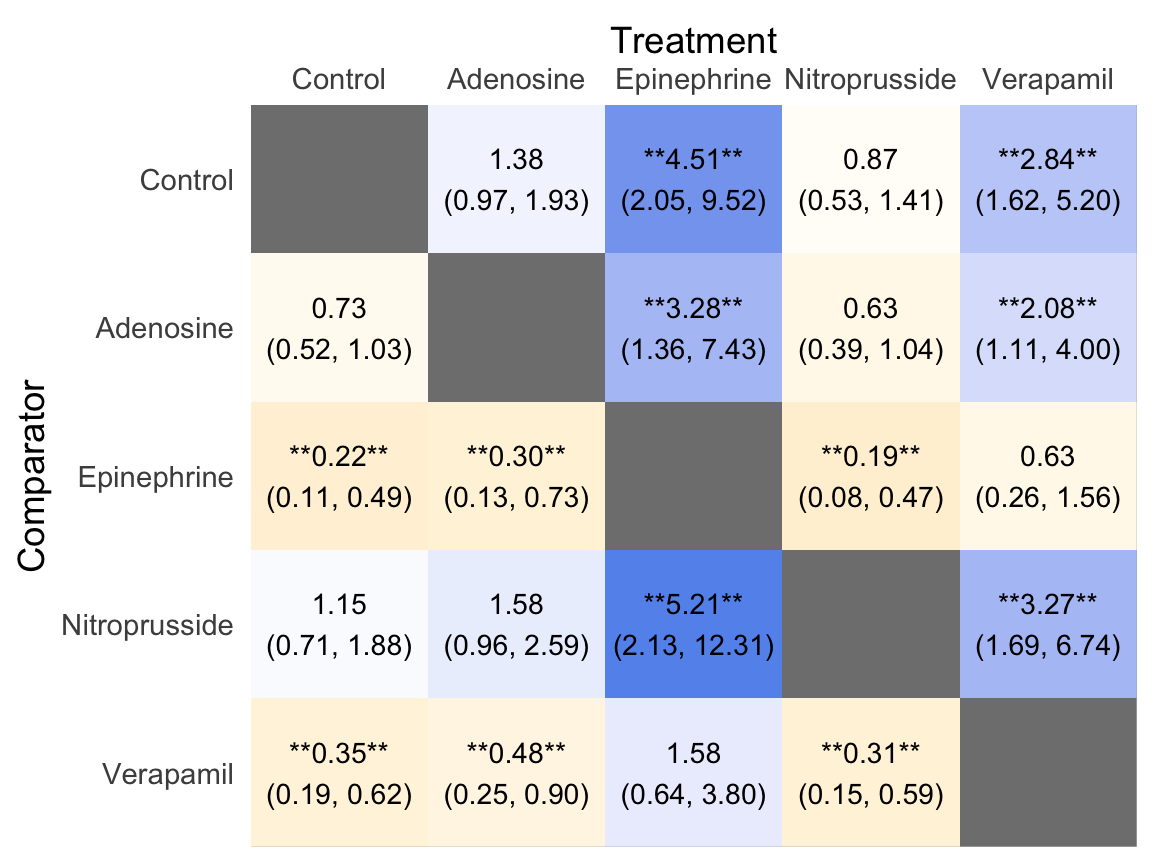
**

**Figure S20.** Bayesian Heatmap Showing Pairwise Odds Ratios for Achieving STR.

**
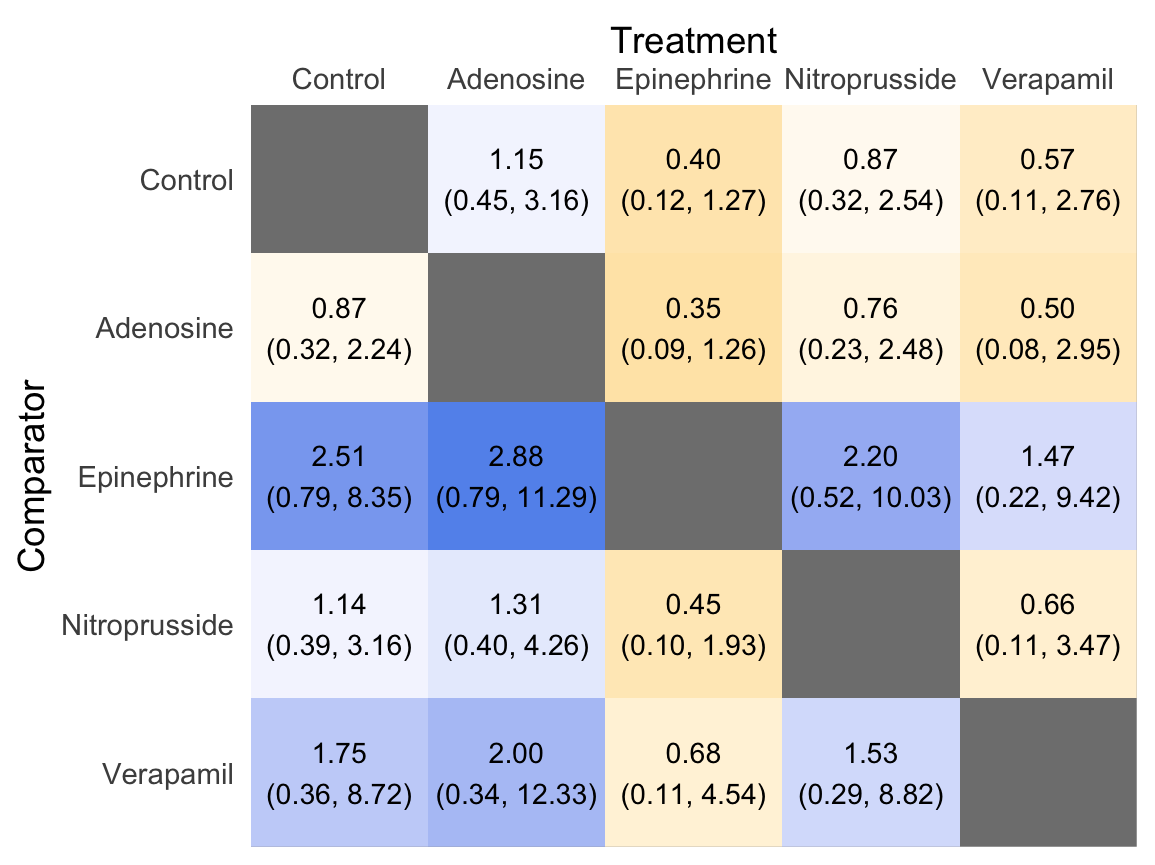
**

**Figure S21.** Bayesian Heatmap Showing Pairwise Odds Ratios for MACE.

**
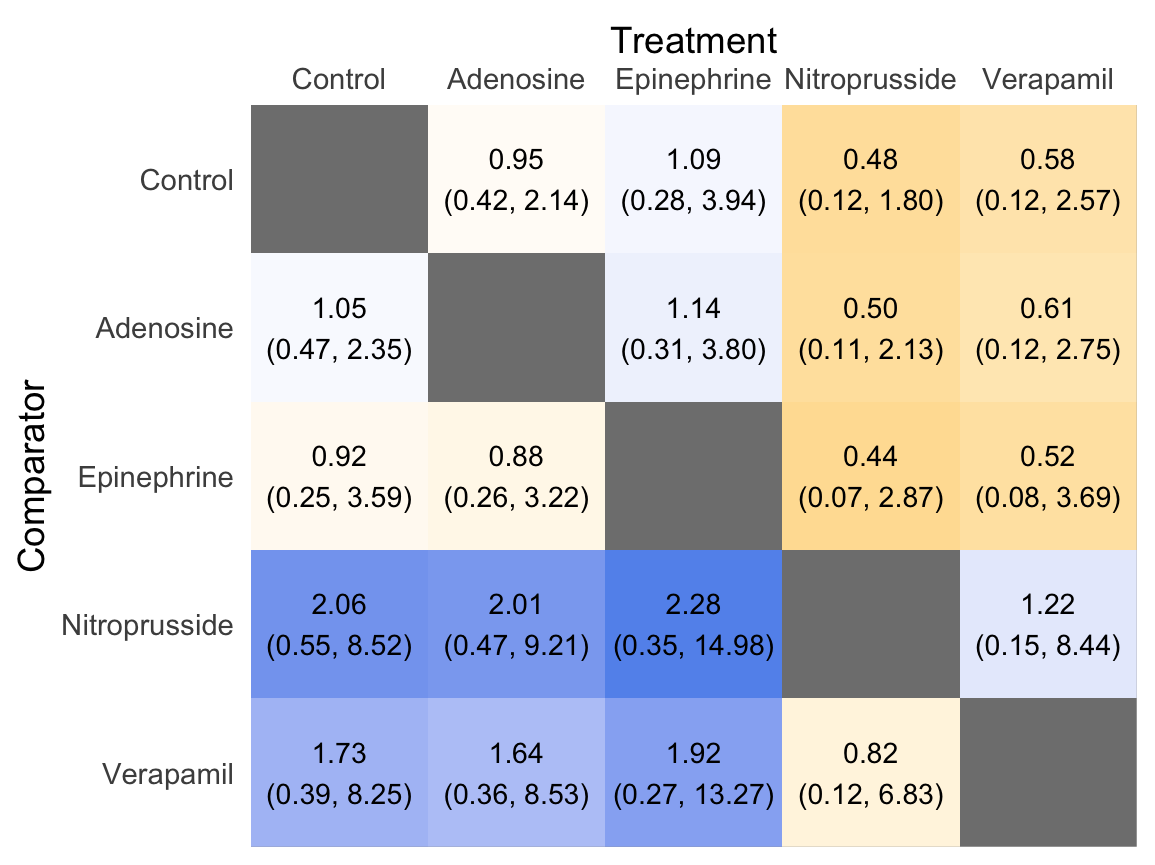
**

**Figure S21.** Bayesian Heatmap Showing Pairwise Odds Ratios for Mortality.

**
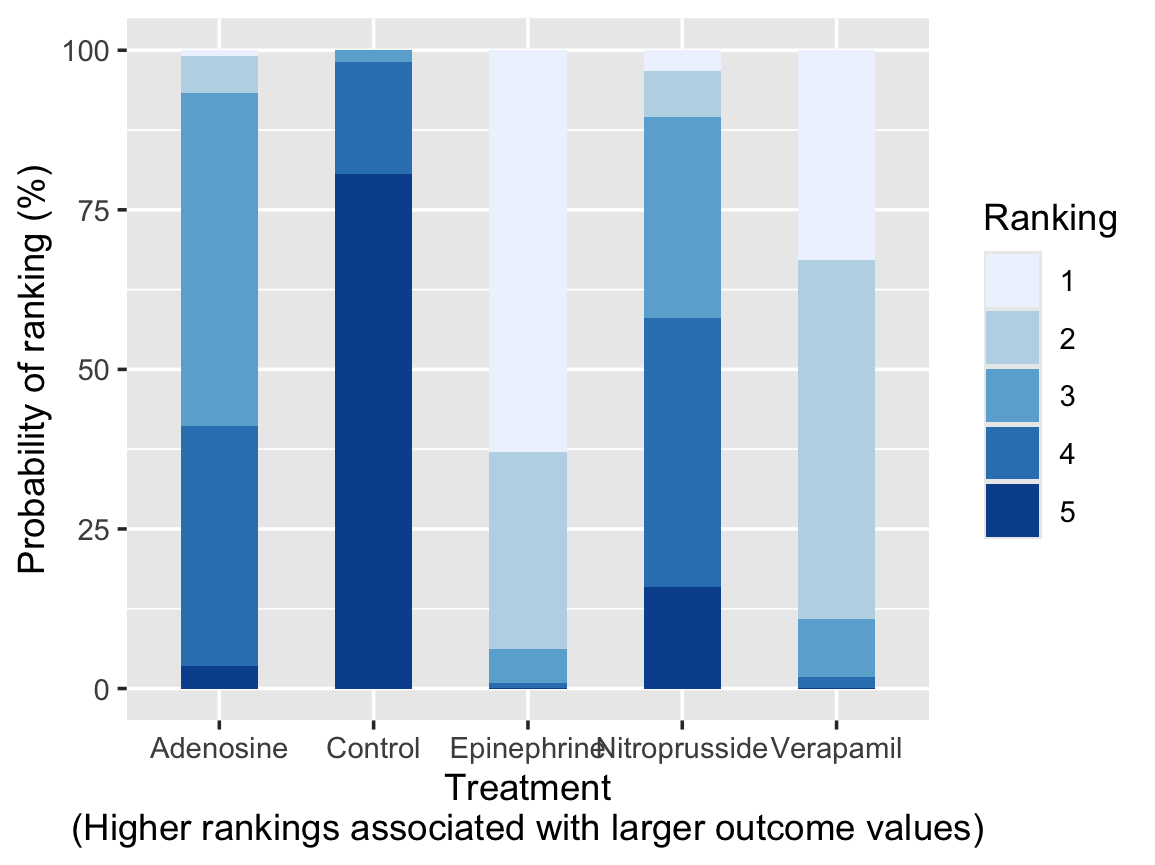
**

**Figure S22.** Rankogram showing the probability of each treatment Ranking 1st to 5th for Achieving TIMI 2-3 Flow.

**
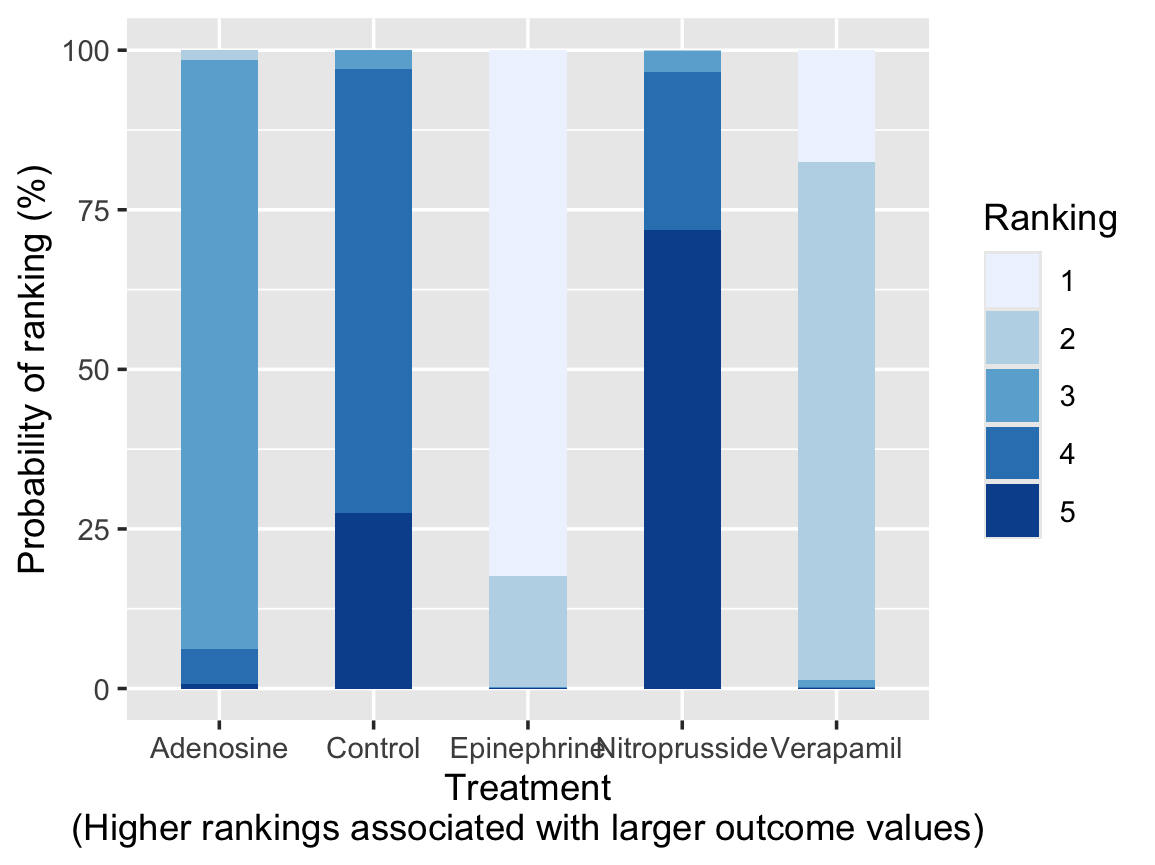
**

**Figure S23.** Rankogram showing the probability of each treatment Ranking 1st to 5th for achieving STR.

**
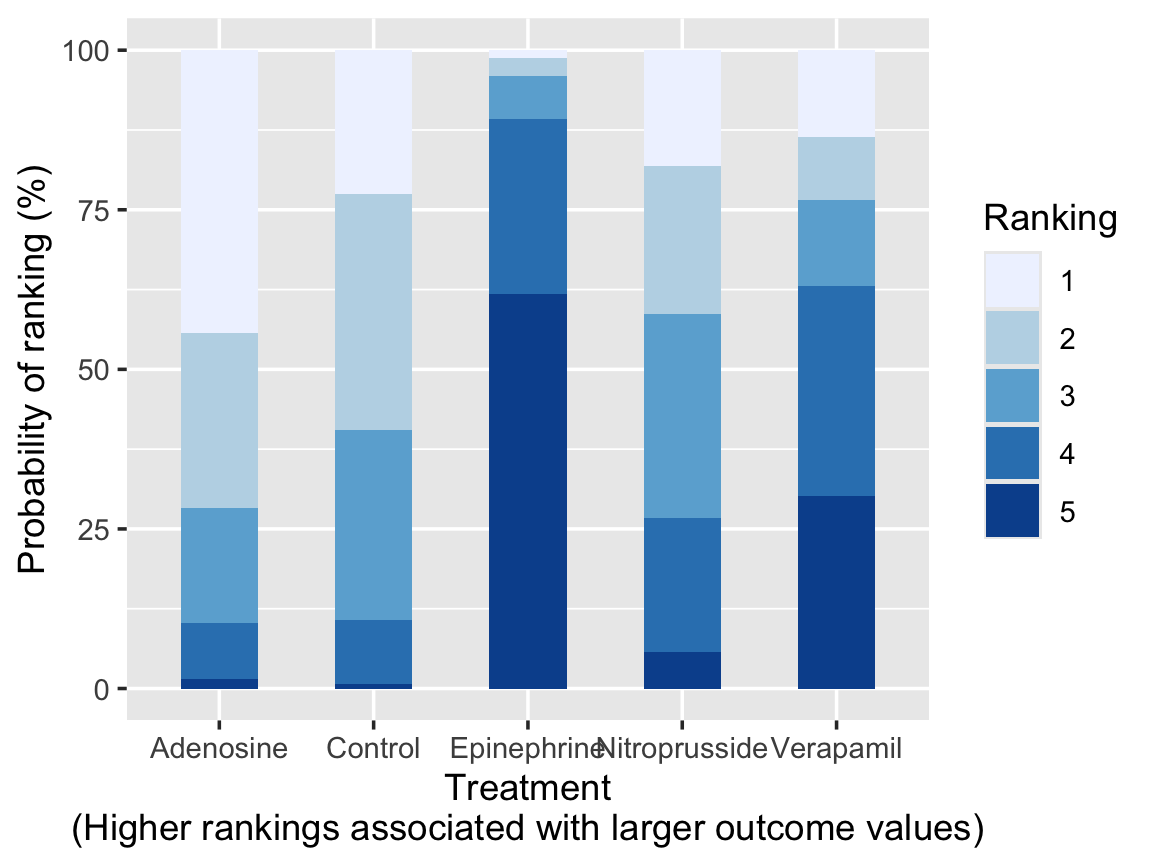
**

**Figure S24.** Rankogram showing the probability of each treatment Ranking 1st to 5th for achieving MACE.

**
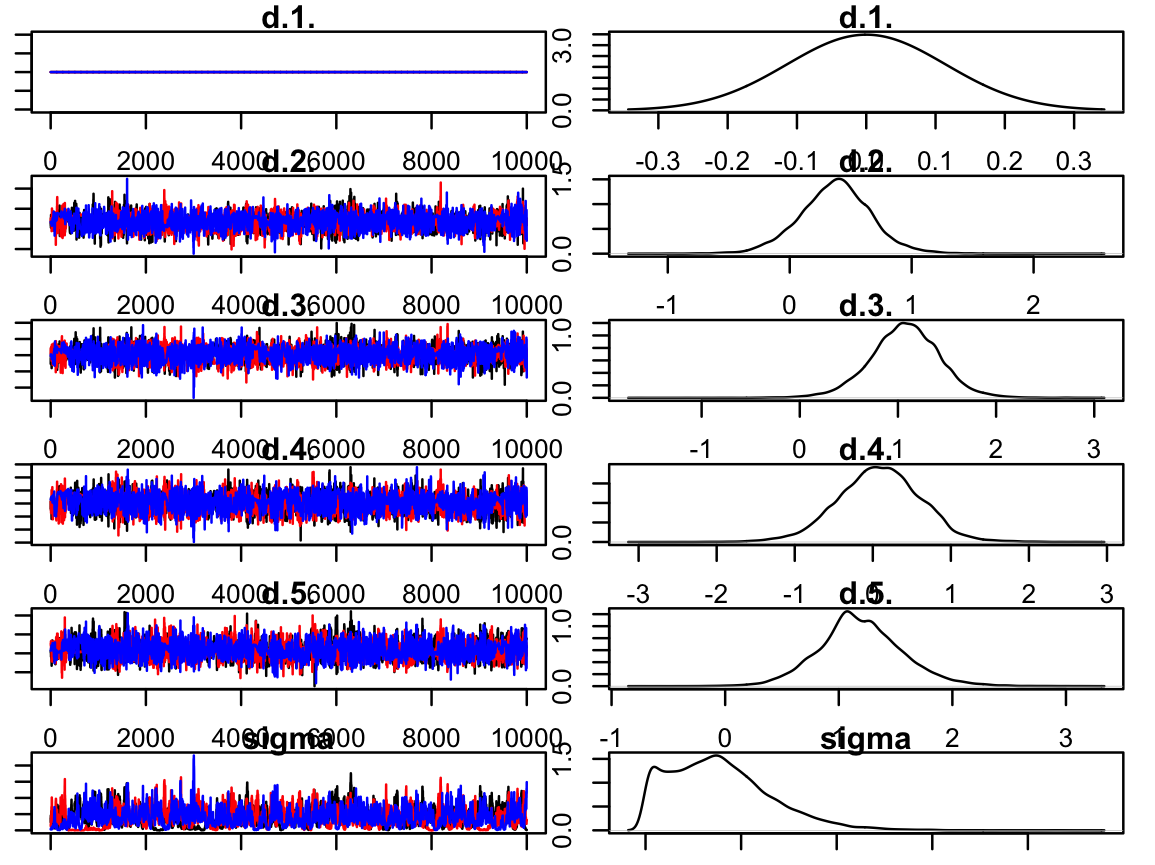
**

**Figure S25.** MCMC trace plots and posterior distributions for treatment effects (final TIMI 3 flow) and between-study heterogeneity.

**
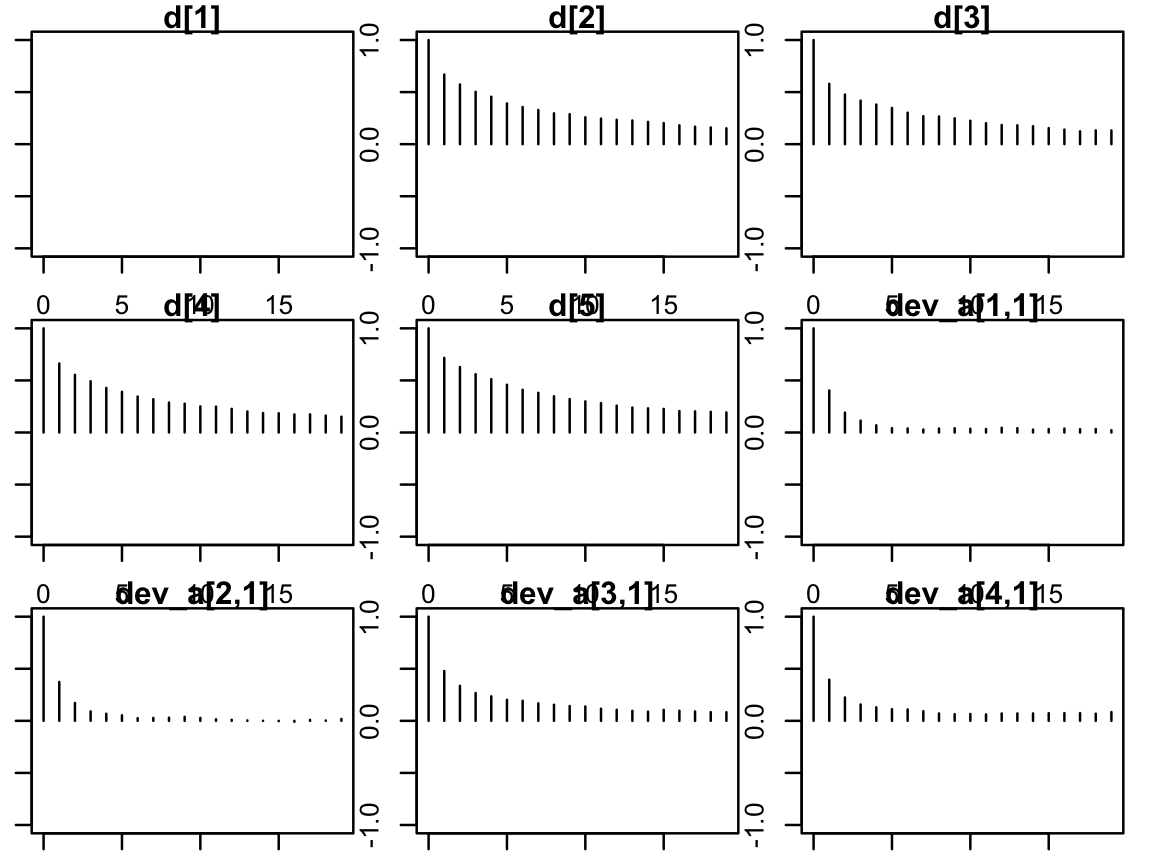
**

**Figure S26.** MCMC autocorrelation plots for treatment effect (final TIMI 3 flow) and model parameters.

**Sensitivity analysis: leave-one-out analysis for the achievement of final TIMI 3 flow**

**
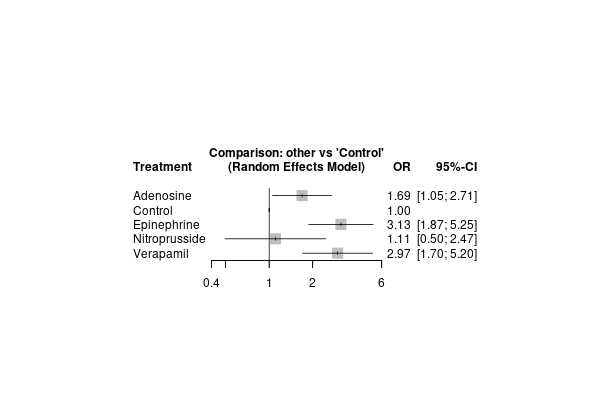
**

Study excluded: Desmet et al.

**
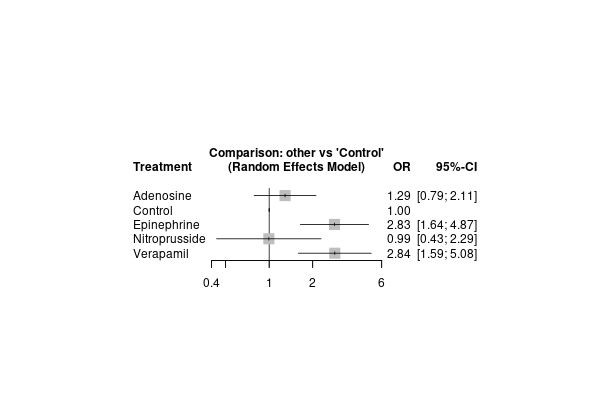
**

Study excluded: Garcia-Dorado et al.


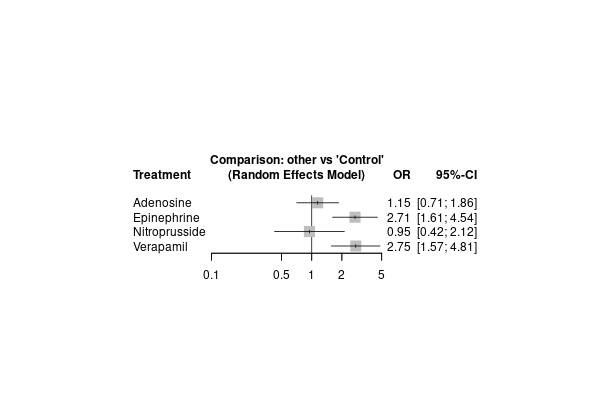


Study excluded: Naghshtabrizi et al.


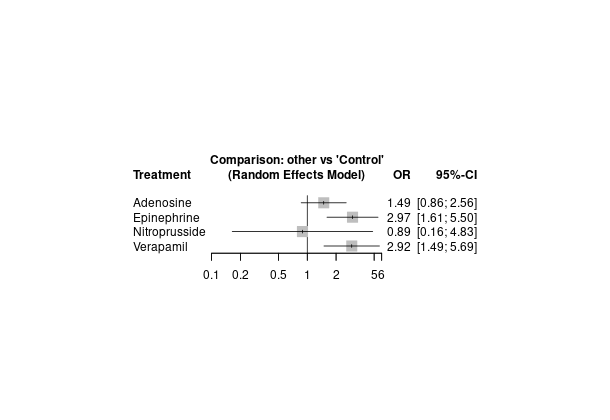


Study excluded: Niccoli et al.


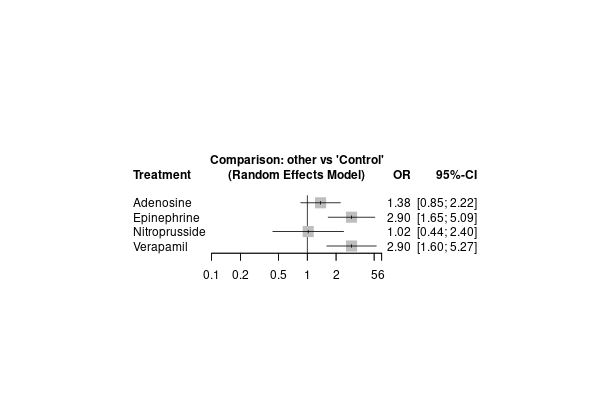


Study excluded: Petronio et al.


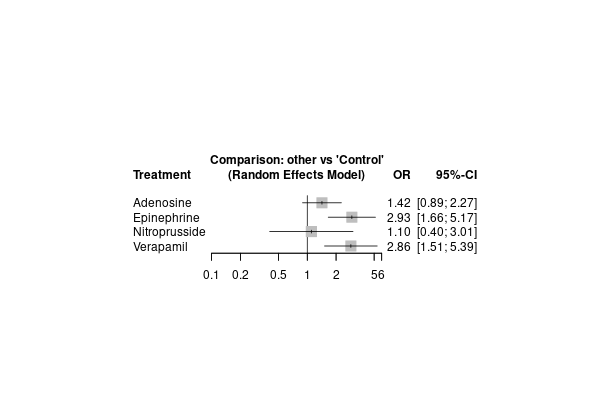


Study excluded: Abdelaziz et al.


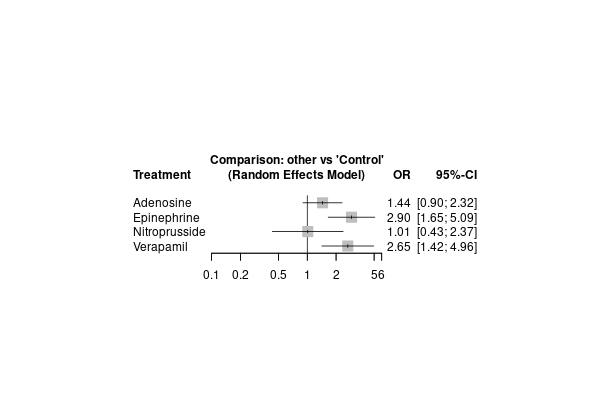


Study excluded: Akturk et al.


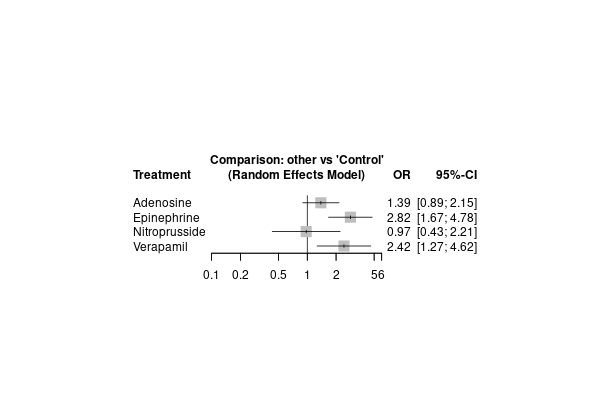


Study excluded: Huang et al.


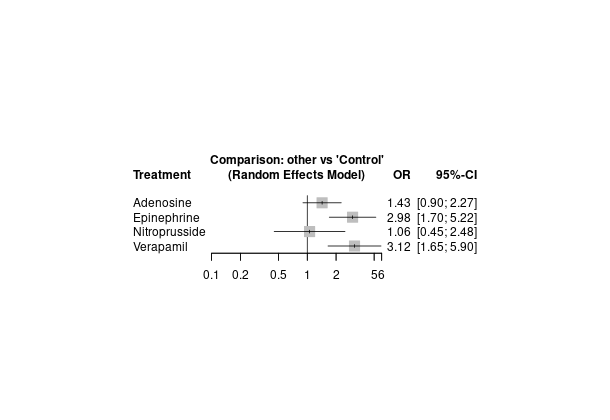


Study excluded: Taniyama et al.

Study excluded: Yassin et al.


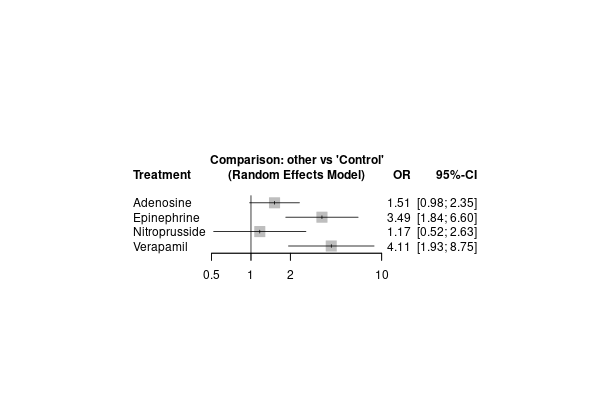


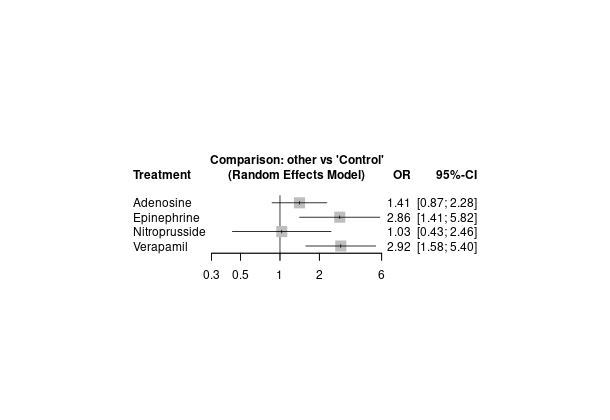


Study excluded: Ryabov et al.


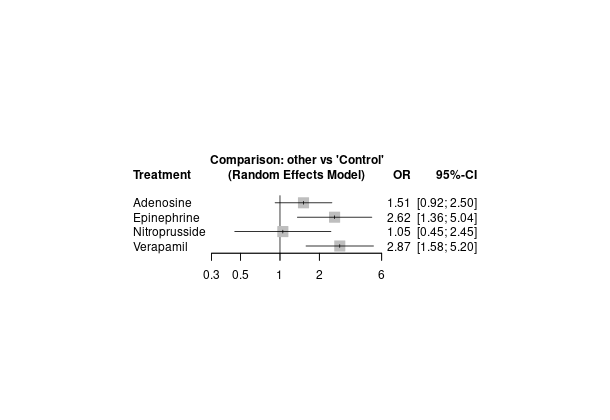


Study excluded: Khan et al.

**Sensitivity analysis: removing the study with high risk of bias**


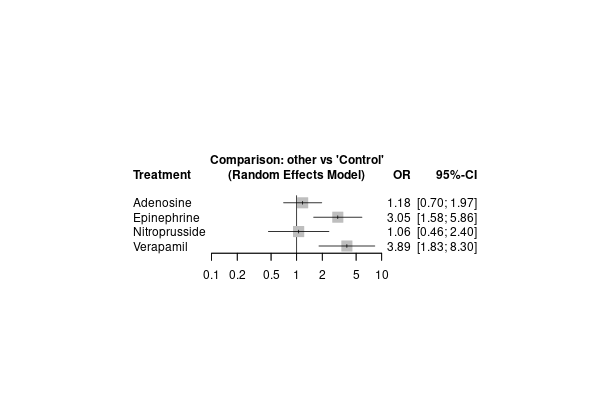


**Figure S27.** Forest plot of odds ratios (ORs) and 95% confidence intervals for achieving final TIMI 3 flow after excluding studies at high risk of bias.


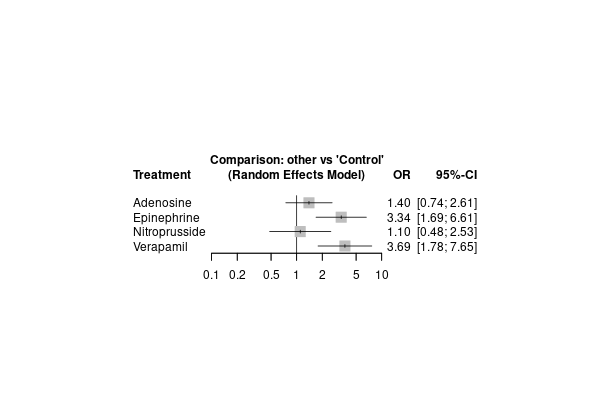


**Figure S28.** Forest plot of odds ratios (ORs) and 95% confidence intervals for achieving final TIMI 2-3 flow after excluding studies at high risk of bias.


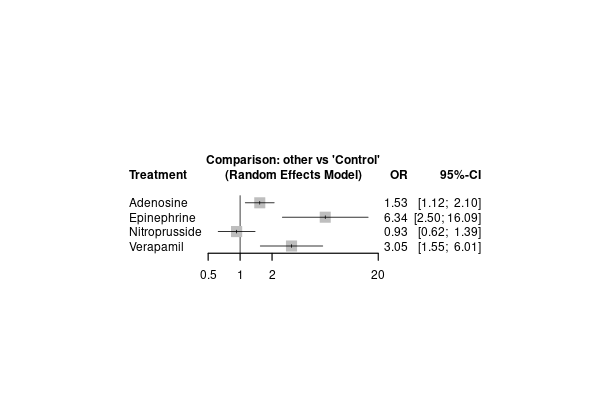


**Figure S29.** Forest plot of odds ratios (ORs) and 95% confidence intervals for achieving STR after excluding studies at high risk of bias.


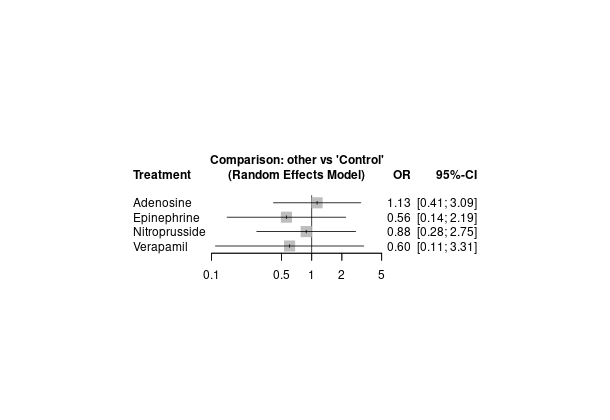


**Figure S30.** Forest plot of odds ratios (ORs) and 95% confidence intervals for MACE after excluding studies at high risk of bias.


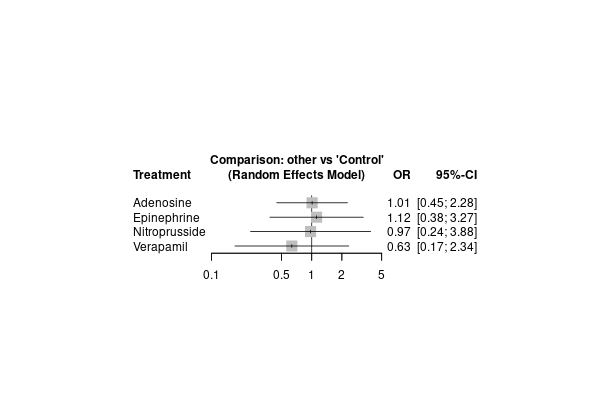


**Figure S31.** Forest plot of odds ratios (ORs) and 95% confidence intervals for mortality after excluding studies at high risk of bias.

**META REGRESSION**

**
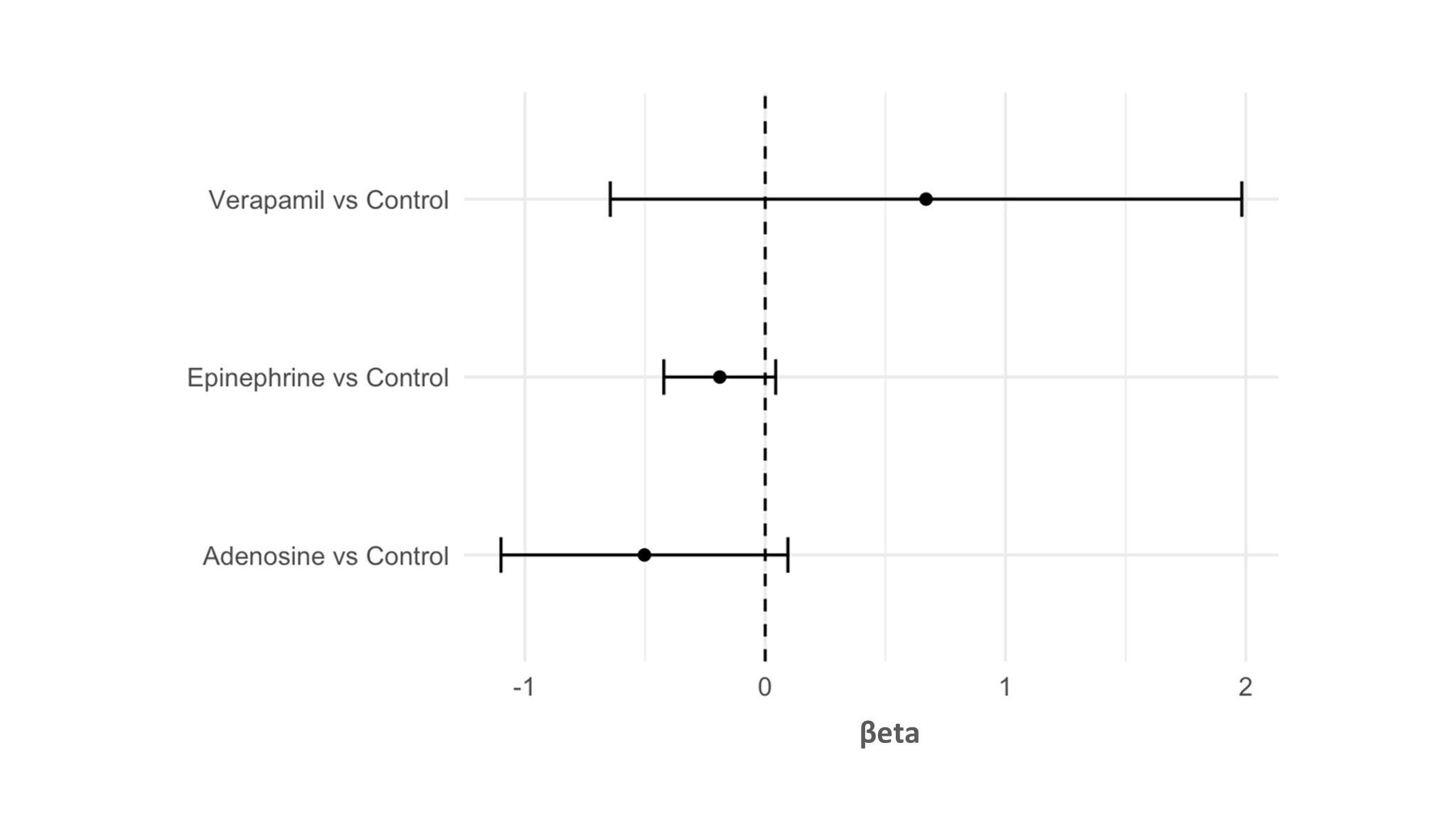
**

**Figure S32.** β Coefficients for the Effect of Age on Final TIMI 3 Flow in a Meta-Regression Analysis.


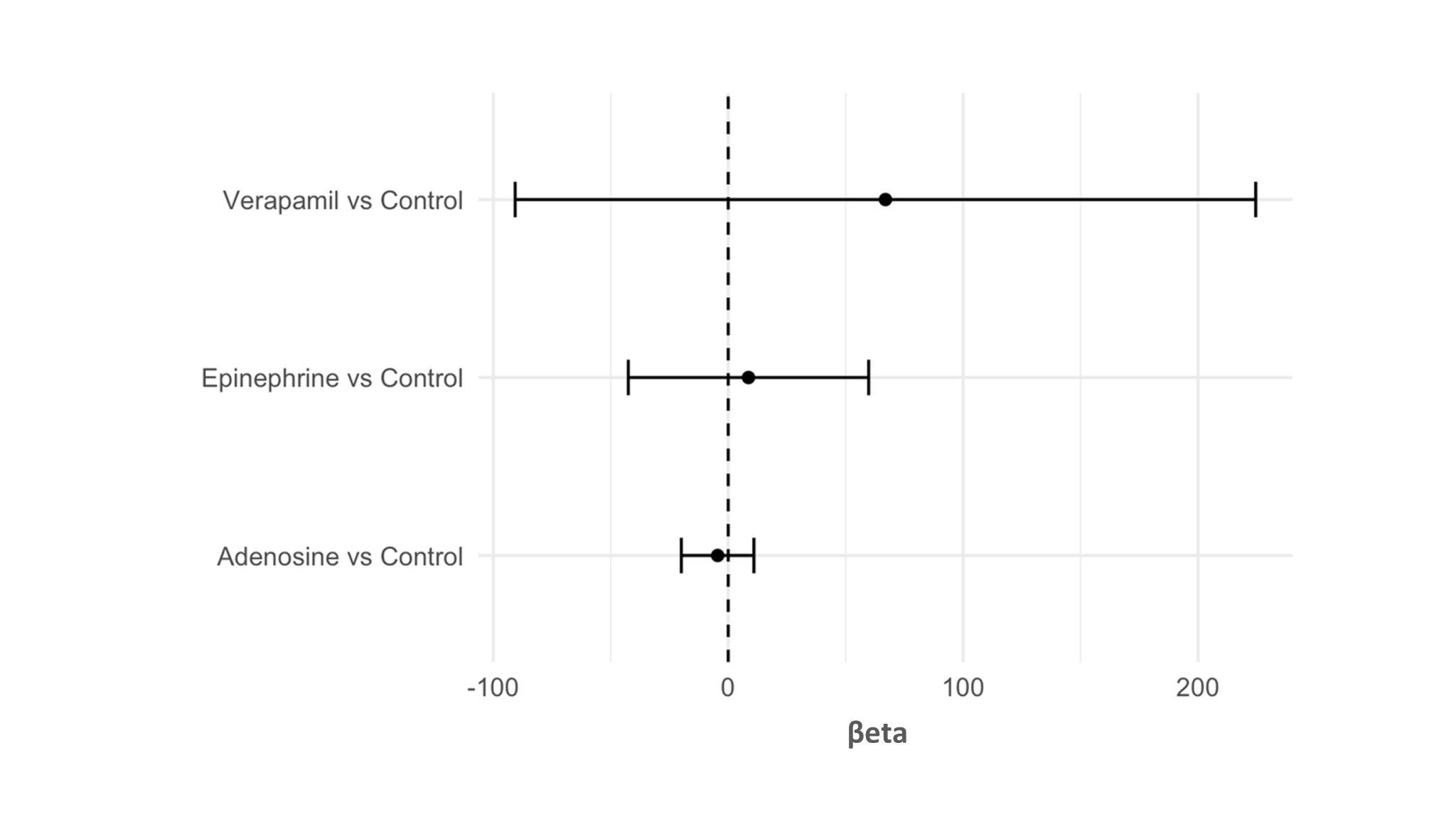
**Figure S33.** β Coefficients for the Effect of Female on Final TIMI 3 Flow in a Meta-Regression Analysis


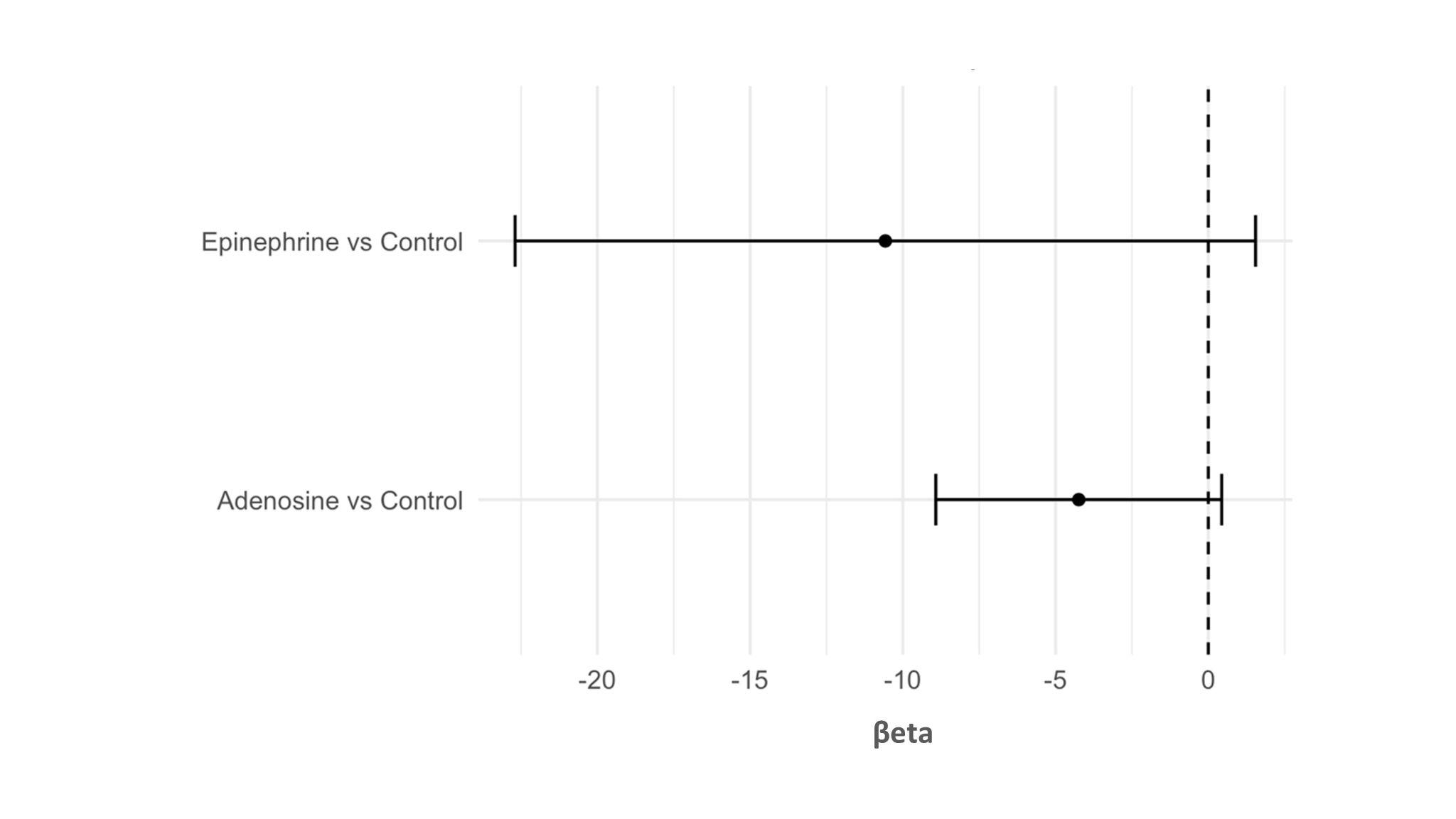


**Figure S34.** β Coefficients for the Effect of Gp IIb/IIIa inhibitors on Final TIMI 3 Flow in a
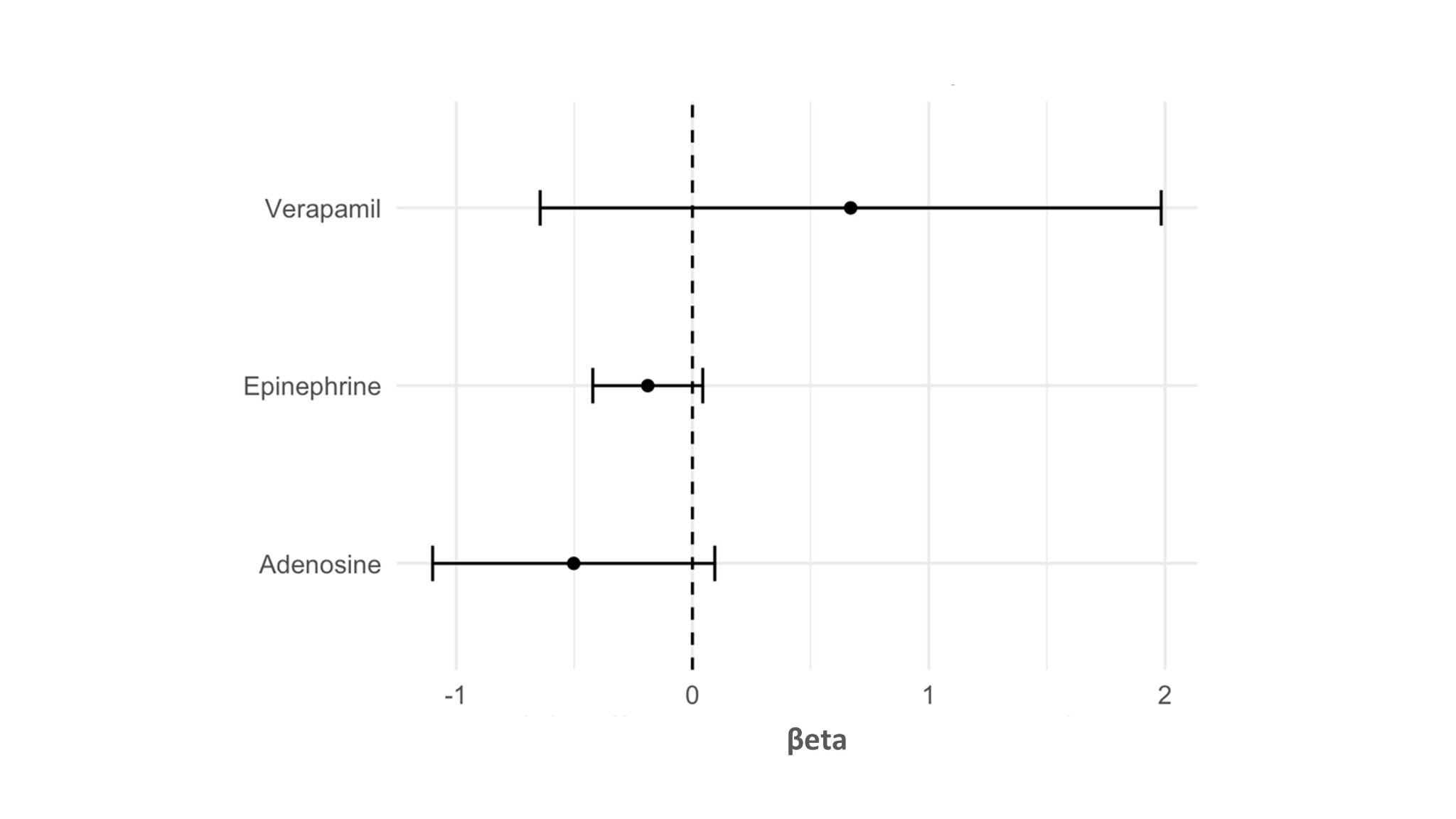
Meta-Regression Analysis.

**Figure S35.** β Coefficients for the Effect of thrombectomy on Final TIMI 3 Flow in a Meta-Regression Analysis.

**Figure S36.** Figure X. Contribution matrix showing the relative contribution of direct comparisons to network meta-analysis estimates for the final TIMI 3 flow.
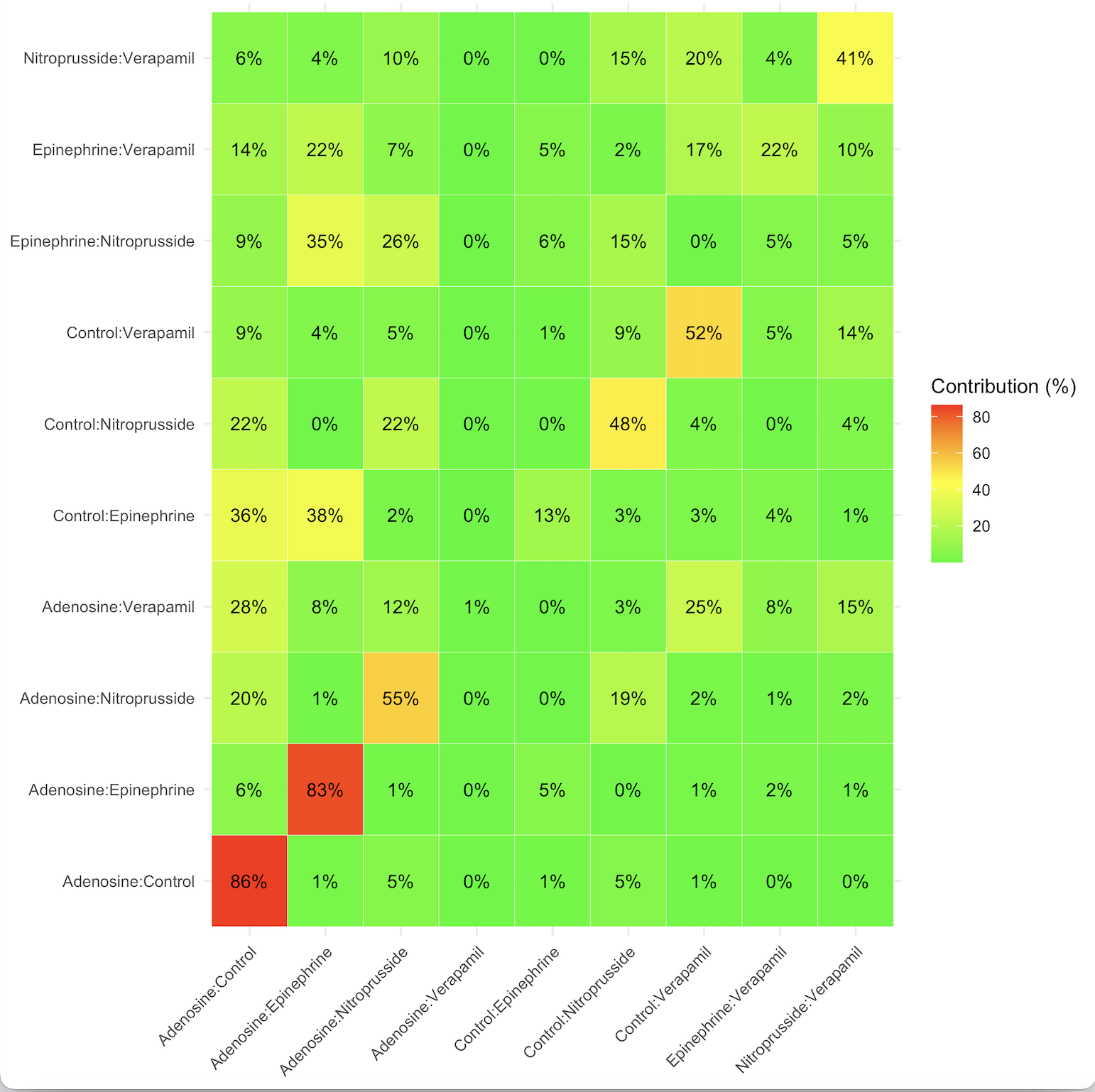

Supplement: Supplemental Material [file mmc1.docx]
